# Parent Speech in Free Play Is Guided by Infant Attention, But Organized by Object Familiarity

**DOI:** 10.1111/infa.70089

**Published:** 2026-05-04

**Authors:** Anne‐Kathrin Mahlke, Shreya Venkatesan, Nivedita Mani

**Affiliations:** ^1^ RTG 2906 “Curiosity” Georg August University Göttingen Göttingen Germany; ^2^ Psychology of Language Department Georg August University Göttingen Göttingen Germany

## Abstract

Successful coordination of infant attention and parent speech during free play supports infants' language development. Parents' responsive linguistic input reduces uncertainty in label‐referent associations and provides information at moments of infants' increased attention and receptiveness. While infants frequently lead the dyads' focus of attention, parent speech has been shown to scaffold infants' attention toward fixated objects. So far, however, little is known about the qualitative characteristics of parent speech during such interactions, and their effects on infants' attention. Here, we analyzed the role of the content and communicative intent of caregivers' speech to their 18‐month‐old infants (*N* = 31) during free play when infants or parents led an interaction, and when parent speech co‐occurred with infants' sustained attention. Interactions were most likely to be infant‐led, both temporally and topically, and parents' topically aligned, but not misaligned speech was associated with infants' sustained attention. Qualitative analysis of speech types revealed mostly object‐focused speech in interactions with familiar objects, but a broader range of speech types in interactions with novel objects. We explain this pattern by suggesting that speech in interactions with familiar objects is structured by shared experience, whereas the lack of common ground with novel objects potentially induces parents to use more varied speech to engage their infants.

## 1 Introduction

Infant language acquisition is a quintessentially collaborative endeavor. Research has long focussed on the importance of a proficient interaction partner who provides sufficient and high quality linguistic input in infants' language development (Anderson et al. 2021; Coffey and Snedeker 2025; Rowe et al. 2017). Other studies have emphasized infants' active role in their learning progress, showing that infants in their second year of life actively choose when and which objects or events in their environment to attend to, and whom to learn from (c.f. Mani and Ackermann 2018, for a review; Ackermann et al. 2020; Begus and Southgate 2012). Combining these lines of research indicates that successful language development relies on the dynamic interplay between infants' active information seeking and caregivers' rich linguistic input. Indeed, studies find enhanced learning outcomes for object functions, visual properties, and labels when caregivers contingently provide information about objects that infants explicitly point to (Begus et al. 2014; Lucca and Wilbourn 2019). Similarly, the extent to which caregivers provide information in response to infants' displays of interest, especially early in the second year of life, predicts infants' vocabulary size and other language milestones (Tamis‐Lemonda et al. 1998; Tamis‐LeMonda et al. 2004, 2001). Against this background, the current study will examine the back‐and‐forth of caregiver‐child interactions, with a particular focus on how parent speech interacts with infant visual attention. In particular, we examine the characteristics of parent speech that precedes or follows infant visual attention, or is associated with infants' extended visual attention.

### 1.1 Parent Speech May Guide Infant Attention

Previous work has highlighted the egocentricity of infant attention in caregiver‐child interactions. During free play, 1 to 2‐year‐old infants tend to pay little attention to their caregivers' faces and, therefore, may not always be aware of their caregivers' focus of (visual) attention (Chen et al. 2020; Madhavan et al. 2026). Madhavan et al. (2026), for instance, found that episodes of caregiver‐infant joint attention are overwhelmingly more likely to be initiated by infants' gaze shifts relative to caregiver gaze shifts, that is, by the caregiver joining the focus of their child's visual attention. Similarly, C. Yu et al. (2021) found that 19‐month‐old infants are equally likely to fixate an intended or incorrect object upon hearing parents label an object in a clustered scene. In contrast, when parents join infants' attentional focus with their gaze and speak about the focussed object, the provided information is contingent with infants' attention (Chen et al. 2021; Elmlinger et al. 2019; Goupil et al. 2024; Madhavan et al. 2026). These findings suggest that caregiver‐infant dyads are much more likely to successfully coordinate their attention when caregivers follow infants' focus of attention, compared to when caregivers try to redirect infants' attention to a certain object.

However, it may at times be necessary for parents to direct their infant's attention toward a different object in the environment. For example, Madhavan et al. (2026) and Goupil et al. (2024) found that, while playing with novel and familiar objects, 14‐ and 18‐month‐old infants prefer looking at and interacting with familiar objects. Furthermore, Madhavan et al. (2026) reported that overall, parents labeled familiar objects more often than novel objects (see also Chen et al. 2021), likely because they followed their infants' attention allocation. Thus, infants may receive little input on novel objects, potentially slowing down their progress in acquiring novel information. While parents could redirect their child's attention to novel objects, it is currently unclear when and how parents successfully do so. One potentially powerful tool for guiding and redirecting infants' attention may be parent speech.

Few studies have examined the role of parent speech in caregiver‐child interactions, especially with regards to the characteristics of parent speech that follow, scaffold, or lead infant attention. When parents were explicitly instructed (via earphones) to deflect their infants' attention to a different object, Deák et al. (2008) found that explicitly directive actions, such as pointing and directive verbalizations—relative to merely calling the child's name—resulted in shared attention to the intended object in both 15‐ and 21‐month‐old infants, with older infants following directive verbalizations more often than younger infants. Indeed, multisensory communication between infants and their caregivers, including speech and manual actions, has been shown to be particularly associated with infants' extended visual attention (Suanda et al. 2016; Suarez‐Rivera et al. 2019), and C. Yu and Smith (2013) found that both caregivers and 13‐month‐old infants coordinate visual attention through their own and their partners' object manipulations during free play. In a later study, Chang and Deák (2019) found that mothers were more likely to use descriptive utterances and utterances containing an object label when their 12‐month‐old infants were looking toward one or more toys, whereas they used more imperative utterances, utterances referring to the infant's attentional state, and utterances containing the infant's name when infants were looking away from them and the toys. Thus, parents use different types of utterances in response to the attentional states of their infants, for example providing information when infants are engaged with objects and attention‐directing language when infants seem to be distracted.

However, these studies provide limited insight into the dynamics of parent speech in caregiver‐child interactions. While Chang and Deák (2019) showcase the characteristics of speech that parents use to try and guide their child's attention, they did not consider whether such attempts at redirection were followed by attentional shifts. Conversely, while Deák et al. (2008) examined the characteristics of parent speech that preceded shifts in infant attention, they did not consider infants' attentional state before parents' speech. Naturalistic interactions, however, are dynamic situations made up of a constant back and forth, where each participant's behavior is tightly related to previous and subsequent actions by the other participant. Against this background, the current study will examine both the characteristics of parent speech that precede infant gaze, as well as the characteristics of speech parents produce when following infant gaze in a free play interaction.

### 1.2 Parent Speech May Sustain Infant Attention

The positive effect of parents' responsive speech on infant learning is likely also related to infants' pre‐existing attention for a given object. In the second year of life, infants' bids for information are often made up of visual attention, pointing, or babbling (Karadağ et al. 2024). It has also been argued that gazing at, handling of, or babbling toward an object signals an increased state of attention and a “readiness to learn” (Goldstein et al. 2010) in the second year of life, during which infants are especially receptive to new information (Ackermann et al. 2020; Goupil et al. 2024). Indeed, several studies suggest that parent speech co‐occurring with infants' attention extends infants' attention to an object (henceforth, sustained attention, SA), especially in the second year of life (Peters and Yu 2020; Schroer and Yu 2022; Suanda et al. 2016; Suarez‐Rivera et al. 2019). Relatedly, caregiver involvement during free play has been shown to coincide with SA in both infants and older children between 6 and 36 months (Gardner‐Neblett et al. 2016). Furthermore, parents' responsive reactions to younger 9‐month‐olds’ SA support word learning both in the short and long term (C. Yu et al. 2019; C. Yu and Smith 2012), and infants' SA has been shown to predict a variety of long term cognitive outcomes (Johansson et al. 2015; Kannass and Oakes 2008; Ruff and Lawson 1990). Taken together, these findings suggest that parents' responsive speech helps sustain infants' attention to objects further, potentially supporting infants' deeper processing of the information provided.

So far, however, little is known about the characteristics of parent speech following infant SA or of the parent speech that precedes and potentially supports infant SA. Some evidence suggests that speech co‐occurring with infant SA tends to be informative. Peters and Yu (2020) found that 19‐month‐old infants' increased SA was related to caregivers' referential compared to non‐referential speech, and to speech describing object features. However, this study did not consider infants' attentional state prior to the utterance. Indeed, the authors only analyzed utterances where parent speech was topically aligned to infant gaze, but not utterances where parents were speaking about an object that infants were not already attending to. Thus, it remains unclear how parent speech characteristics change depending on infants' attentional states, and how different speech characteristics interact with infants' SA during dynamic, naturalistic interactions.

### 1.3 Current Study

The current study examines parent speech characteristics, particularly speech content and communicative intent, within the temporal dynamics of caregiver‐child interactions with novel and familiar objects. We will explore the linguistic characteristics of parent speech to infants when their speech is contingent with their infants' focus of visual attention and when it is not. Furthermore, we examine the characteristics of parent speech associated with changes in infants' attention to objects, i.e., the characteristics of speech that co‐occur with sustained or shortened infant attention.

Speech may be a particularly powerful tool which parents have at their disposal to scaffold and direct their child's attention. It is, therefore, surprising that little is known about the linguistic characteristics of parent speech to children in caregiver‐infant interactions. Investigating the complex interplay between parents' speech and infants' attention in an ecologically valid setting provides insights into the characteristics of speech which may be related to shifts or extensions of infants' attention toward objects in their environment. This may further inform strategies parents and caregivers can employ to explicitly support their infants' information processing and learning.

Thus, our overarching research questions examined (a) whether caregiver‐child interactions were more likely to be led by infant gaze or parent speech, that is, an instance of infant gaze followed by parent speech (infant‐led) or a parent utterance followed by an infant gaze toward an object (parent‐led) and (b) in cases where parent speech followed infant gaze, the extent to which parent speech was correlated with sustained infant gaze toward an object. We examined each of these research questions in terms of (i) the topical alignment of parent speech and infant gaze, that is, whether parents were talking about the object that the infant was also looking at or a different object, (ii) the novelty of the object the infant was attending to and (iii) the quality of speech—in terms of both the content and communicative intent of utterances—that parents produced in different kinds of interactions.

To this end, we reanalyzed a set of video‐recordings of 18‐month‐old infants and their parents playing with different toys that had been collected for a previous study (Madhavan et al. 2026). We extracted all instances of infants' looks toward an object as well as transcribed and coded parent utterances throughout the interaction. We coded parent utterances—in keeping with coding systems proposed by Chang and Deák (2019) and Peters and Yu (2020)—on two levels. The first level coded for the content of parent speech, including referential speech, social references, speech containing object information, and situational speech (e.g., comments, acknowledgments, recasts, exclamations, play). The second level coded for the communicative intent of the utterance, containing imperatives, declaratives, and four different types of questions (descriptive, directive, informative and pedagogical).

The research questions examined in the current study and our hypotheses are presented in Table 1, which also includes the syntax of the models examining these research questions (which are explained in further detail below) and the main results.

**TABLE 1:** Overview of the study's research questions, hypotheses, models, and results.

Research questions	Hypotheses	Model syntax	Result summary
Leader‐follower dynamics			
RQ 1.1a: Are caregiver child interactions more likely to be led by infant gaze or parent speech?	H 1.1a: Given that speech is a particularly powerful tool that parents have at their disposal, we hypothesize that caregiver‐child interactions were more likely to be initiated by parent speech than by infant gaze	Model 1.1: *leader ∼ novelty* *+* *z.age* *+* *z.offset* *+* *(novelty || ID)* *+* *(1|object)*	Caregiver‐child interactions were more likely to be initiated by infant gaze than by parent speech
RQ 1.1b: Are infant‐led and parent‐led interactions more likely to focus on novel or familiar objects?	H 1.1b: We expected infant‐led interactions (Goupil et al. 2024; Madhavan et al. 2026) to focus on familiar objects, but parent‐led interactions to focus on novel objects (capturing caregivers' attempts to introduce novel objects to their infants)		No evidence that infant‐led or parent‐led interactions were more likely to focus on familiar objects
RQ 1.2a: Are infant‐led and parent‐led interactions more likely to be topically aligned or misaligned?	H 1.2a: Given previous findings indicating that infants are equally likely to fixate a correct or incorrect referent upon hearing parent speech (C. Yu et al. 2021) and that parent speech is contingent in response to infant gaze (Abney et al. 2020; Chen et al. 2021; Elmlinger et al. 2019; Goupil et al. 2024), we expected infant‐led interactions to be more topically aligned than parent‐led interactions	Model 1.2: *leader ∼ alignment* *+* *novelty* * *(referential* *+* *social* *+* *situational* *+* *object information)* *+* *z.age* *+* *z.offset* *+* *(alignment* *+* *novelty || ID)* *+* *(alignment || object)*	Topically aligned interactions were more likely to be infant‐led than parent‐led
RQ 1.2b: Are infant‐led and parent‐led interactions characterized by differences in the content of parent speech?	H 1.2b: We expected parents to provide more referential and informative speech in infant‐led than in parent‐led interactions, illustrating parents' attempts to scaffold infant gaze		Parent‐led interactions concerning *familiar objects* contained more referential speech than infant‐led interactions
			Parent‐led interactions concerning *novel objects* contained more referential, social, and informative speech than infant‐led interactions
RQ 1.3: Are infant‐led and parent‐led interactions characterized by differences in the communicative intent of parent speech?	H 1.3: We expected parents to provide more declaratives, pedagogical, and descriptive questions in infant‐led than in parent‐led interactions, illustrating parents' attempts to scaffold infant gaze. In parent‐led interactions, we expected parents to use more directive and attention‐grabbing speech (i.e. imperatives and directive questions)	Model 1.3: *leader ∼ alignment* *+ novelty* * *intent* *+* *z.age* *+* *z.offset* *+* *(alignment* *+* *novelty || ID)* *+* *(alignment || object)*	Results were inconsistent across different models. We, therefore, treat these findings with caution
Sustained attention			
RQ 2.1a: Is topically aligned parent speech more associated with sustained infant gaze than topically misaligned speech?	H 2.1a: In keeping with the purported scaffolding effect of parents' speech on infants' attention, we expected topically aligned parent speech to co‐occur with more extended infant gaze than topically misaligned speech	Model 2.1: *SA ∼ alignment* *+* *novelty* *+* *z.age* *+* *z.offset* *+* *(alignment* *+* *novelty | ID)* *+* *(alignment |object)*	Topically aligned parent speech was associated with infants' extended attention, while topically misaligned speech was associated with shorter bouts of attention after speech onset
RQ 2.1b: Is parent speech about familiar objects more associated with sustained infant gaze than parent speech about novel objects?	H 2.1b: We expected parent speech about familiar objects to co‐occur with more extended infant gaze than parent speech about novel objects as the referent of the utterance is unambiguous for the infant, while parent speech about novel objects is referentially more ambiguous		There was no significant effect of object familiarity on infants' sustained attention
RQ 2.2: Are there differences in the characteristics of parent speech in terms of its content that are associated with infants' sustained gaze?	H 2.2: We expected parent speech to be associated with extended infant attention especially when parents use referential speech and speech containing object information (Peters and Yu 2020). On the other hand, we expected infants' gaze toward an object to decrease when parents speak about a different object and use more directive and attention‐grabbing speech (Chang and Deák 2019)	Model 2.2: *SA ∼ alignment* *+* *novelty* * *(referential* *+* *social* *+* *situational* *+* *object information)* *+* *z.age* *+* *z.offset* *+* *(alignment* *+* *novelty | ID)* *+* *(alignment || object)*	While some results were inconsistent across models, informative speech about *familiar objects* was associated with extended infant gaze
RQ 2.3: Are there differences in the characteristics of parent speech in terms of its communicative intent that are associated with extended infant gazes?	H 2.3: We expected infants' gaze toward an object to decrease when parents speak about a different object than the infant is attending to and use more directive and attention‐grabbing speech (Chang and Deák 2019)	Model 2.3: *SA ∼ alignment* *+* *novelty* * *intent* *+* *z.age* *+* *z.offset* *+* *(alignment* *+* *novelty | ID)* *+* *(alignment | object)*	Parents' use of directive and informative questions was associated with extended sustained attention to *novel objects* after speech onset

## 2 Methods

### 2.1 Ethics

The present study adhered to the principles outlined in the Declaration of Helsinki. Before any recording or data collection, parents provided informed consent for their and their infants' participation. Approval for all procedures involving human subjects was received from the institutes' ethics committee. Each child received a children's book as a token of appreciation for their participation.

### 2.2 Participants

The analyses described here were conducted on a corpus of video recordings collected for a study investigating leading‐following interactions based on parents' and infants' gaze (Madhavan et al. 2026). Thirty‐one parent‐infant dyads (16 boys, 15 girls; age range: 14–23 months, *M*_age_ = 17.74, SD_age_ = 2.92; 25 mothers, 6 fathers) were recorded during a free play session with four different toys. All infants grew up in German‐speaking monolingual families, were born full‐term, and had no diagnosed developmental disorders. All dyads were recruited from a database managed by the laboratory, containing details of predominantly Caucasian parents with at least undergraduate level educational attainments. Twenty additional dyads were excluded due to infants' fussiness (i.e., infants being unwilling to sit at the table opposite their parent, *n* = 10), because the child grew up bilingual (*n* = 1), all toys were familiar to the infant (hence not fulfilling the study criteria of playing with novel and familiar toys; *n* = 3), or the infant refused to play with any of the toys for at least 2 minutes (*n* = 6). As this study was conducted using pre‐existing data, no power analyses were run to predetermine the sample size. However, we calculated model complexity for all models to ensure model adequacy (see Data Analysis section below).

### 2.3 Stimuli

Small plastic toys were created for the study. Eight objects from two object categories—animals and vehicles—were chosen so that four were typically familiar and four were typically unfamiliar to young children (based on the WordBank (Frank et al. 2017) and CHILDES (Szagun 2001) corpora). For the familiar objects, *cat [Katze]*, *bear [Bär]*, *car [Auto]*, and *bus [Bus]* were chosen, for novel objects *iguana [Leguan/Eidechse/Echse/Gecko/Dino]*,^1^ *seal [Seehund/Robbe/Seekuh/Walross]*, *submarine [U‐Boot]*, and *wagon [Kutsche/Anhänger/Schlitten/Wagen/Planwagen]*. They were 3D‐printed in white and painted in different colors to make them distinguishable on video. The objects had an average size of 167.7 cm3, so they could be held by an 18‐month‐old, but also remained visible when held by an adult.

### 2.4 Procedure

Dyads were recorded playing with four objects, two typically familiar to the infant, and two novel. Before the play session, parents were asked to choose two toys each from two different boxes (one containing the familiar, and the other the novel objects). They were not informed that the objects differed in familiarity for their children, and were asked whether their children knew the chosen objects only after the play session. Thus, we ascertained infants' individual knowledge states and coded the objects accordingly in all analyses. During the play session, dyads were sat at a small table opposite each other, and parents were instructed to play with the objects as they would at home. Two cameras were set up so that infants' gaze and touch, and parents' touch and speech during the interaction could be recorded. Parents wore head‐mounted eye‐trackers for the study, and the resulting video recordings were combined with the recordings from the static cameras for coding. Before the play session, the chosen toys were hidden in a box until the cameras were synchronized by hitting a rattle on the box. The toys were then revealed and parents and infants played until the infant lost interest after 6 minutes had passed.

### 2.5 Coding

*Gaze*. Infant gaze during the play session was manually coded for the original study (Madhavan et al. 2026) using ELAN (v6.3) by three trained coders. The coding was based on the videos from two stationary cameras and the integrated cameras of parents' head‐mounted eye‐trackers. Gazes of 100 ms and longer were coded for five different regions of interest, that is the four toys in the interaction and the parents' face. For the present study, only gazes toward toys were analyzed. Two coders additionally went through all coding and discrepancies were resolved through a discussion between the coders.

*Speech*. Parent utterances were first transcribed using the whisper‐large model for automatic speech recognition (Radford et al. 2022). As the transcripts showed extensive inaccuracies (on average 34.7% correctly transcribed utterances), timestamps and utterances were subsequently corrected by trained research assistants. All transcriptions were checked by three different coders to ensure accuracy. Two different coders coded the onset and offset of the timestamps of 5 datasets (16%) to check interrater reliability of coding of utterance onset and offset. We had good interrater reliability, with coding of utterance onset differing on average by 7.7 ms, utterance end by 96.3 ms, utterance duration by 88.6 ms. Given that average utterance length was 1220.7 ms, this suggests acceptable overlap in timestamp coding across coders. Speech tokens were considered separate utterances if they were separated by at least 400 ms of silence, following common practice in the field (Peters and Yu 2020; Suarez‐Rivera et al. 2019; C. Yu and Smith 2016). Utterances were further divided into C‐units (communication units), according to the SALT (Systematic Analysis of Language Transcripts; Miller and Chapman 1985) conventions^2^ A C‐unit is an independent clause with its subordinate clauses, or an utterance that is clearly separable by its phonological properties. C‐unit segmentation was conducted by a second coder for five datasets (16%), and comparisons revealed on average 2.8% of utterances segmented differently.

Parents' C‐units were coded using the transcripts and audio‐visual recordings of the interaction. First, we coded the topic of each C‐unit, that is the toy being spoken about in the utterance. Note that the topic did not need to be explicitly referred to in an utterance (by using a noun or pronoun to refer to the object) but had to be clearly recognizable to the coder (e.g., from the speech context or the video). It was coded as *other* if the utterance referred to an object, person, or event outside the play situation, or as *unclear*. Only toy‐related utterances were included in the analysis, and each utterance could be coded as having more than one topic. In such cases, the utterance was treated as a separate entry for each topic. To characterize the different speech types, we used a coding system adapted from those created by Chang and Deák (2019) and Peters and Yu (2020). These systems were created for analyses of the interplay between infant attention and parent speech and overlap substantially with other coding systems (Dave et al. 2018; Gros‐Louis et al. 2006; Lee and Ha 2023; Tamis‐LeMonda et al. 2012).

C‐units in the present study were coded at two levels: a content level and a communicative intent level. Descriptions and examples for all speech types can be found in Table 2 (Speech Content) and Table 3 (Communicative Intent). Two trained coders coded all the data. The first author subsequently went through all coding and discrepancies were discussed and resolved amongst the coders. On the content level, utterances were coded in four different categories. Speech was coded as containing referential speech when it referred to one of the toys by an object name or a pronoun (Peters and Yu 2020; Slone et al. 2023), or a typically associated noise, for example, “meow” (German: “miau”) for the cat (Motamedi et al. 2021; Ota et al. 2018; Tamis‐LeMonda et al. 2012). It was coded as containing social references when containing speech referring to oneself, the partner, both partners together, or a person outside the dyad (Zhang et al. 2025), and as containing object information when parents spoke about object features or actions and activities related to the objects (Peters and Yu 2020). Lastly, utterances were coded as containing situational speech when they contained comments, exclamations, acknowledgments, imitations and recasts, or play utterances (Chang and Deák 2019; Peters and Yu 2020). Utterances could be coded as containing several types of content and were counted as separate entries for each type of content they were associated with.

**TABLE 2:** Utterance types on the content level.

Utterance type	Definition	Examples
Referential	References to one of the toys: Labels, pronouns, noises	“Do you like the *car*?” “Are you driving *it*?” “Where is the *vroom vroom*?”
Social	References to a person: Partner, self, both participants, other (person outside the dyad)	“Want *me* to do it?” “Do *you* know what that is?” “*Daddy* is at home.”
Object information	Object features or activities/actions performed with an object	“This cat is *red*.” “The car is *driving* fast!” “Did you *drop* the iguana?”
Situational	Situation‐related speech: Comments, acknowledgments, imitations/recasts, play sounds, exclamations	“Uhh, this is *cool*!” “*Mhm*”/“*Yes*!” Child: “Cat!”—Parent: “*Yes, the cat is over here*!” “*Oh no*!” “*Oops*!”

**TABLE 3:** Utterance types on the communicative intent level.

Utterance type	Examples
Imperatives	“Look at that!”, “Put it there!”
Declaratives	“That one is green!”, “Everything has fallen down now.”, “This is a submarine.”
Informative questions	“Do you like this one?”, “Do you want to drive the car?”, “Should Mummy hold that?”, “Where did you put it?”
Pedagogical questions	“Which animal is this?”, “What is that one called?”, “What noise does the cat make?”, “Where is this one's tail?”
Descriptive questions	“Is the seal driving the car?”, “Are you driving all over this table?”
Directive questions	“Can you come back here for a moment?”, “Why don't you put this one on top?”

On the level of communicative intent, utterances were coded as imperatives, declaratives, and four different types of questions (Peters and Yu 2020). Previous studies have reported that parents employ question formats for many different communicative intents, such as asking for information, eliciting specific responses, directing infants' behavior, and describing current events (Holzman 1972; Olsen‐Fulero and Conforti 1983; Shatz 1978). Therefore, utterances in question formats were coded separately as: pedagogical questions (questions for which the parents already knew the answers; Y. et al. 2019), informative questions (“real” questions that parents did not know the answer to), descriptive questions (descriptions of actions or events in the play situation in a question format), and directive questions (imperatives in a question format). In the communicative intent level, utterances could only belong to one category at a time.

A second coder coded three randomly selected datasets for topic, speech content, and communicative intent (353 utterances, 10.4% of all utterances) to assess inter‐rater reliability. Topic coding showed substantial agreement (Cohen's *κ* = 0.81, 84%). On the speech content level, all categories demonstrated consistently high reliability, with Cohen's *κ* values ranging from 0.72 to 0.80 and raw agreement between 88% and 95%. For the communicative intent level, coding showed moderate‐to‐substantial agreement (Cohen's *κ* = 0.60, 70%)

### 2.6 Preprocessing

The language coding resulted in a file containing data for each utterance of the parent, coded for whether it contained referential, social, informative and situational speech as well as a communicative intent variable with the different levels described above. We also included the Topic level where each utterance was coded across multiple columns for a maximum of three topics that the utterance could pertain to. The dataset also included the time an utterance began and ended for each utterance as well as data of all infant looks to toys from (Madhavan et al. 2026), including the start and end of the look as well as the object the infant was looking at. From the start times of the gazes and utterances, we coded each row for whether the gaze preceded the utterance (1) or the gaze followed the utterance (0) as our main response variable for Models 1.1, 1.2 and 1.3. Only looks that started during an utterance and utterances that started during a gaze were included in the analysis. We coded the topical *alignment* of each row in terms of whether the parent was talking about the object the infant was looking at (0) or talking about a different object to the object the infant was looking at (1). We coded the *novelty* of the object the infant was looking at based on parent feedback after the play session (1 = familiar, 0 = novel). We also calculated an *offset* measure, which was the absolute difference between the start of speech and the start of gaze (i.e., abs(start.infant.gaze—start.parent.speech)). This measure controlled for how long infants had been looking at an object before a parent utterance began or how long parents had been talking about an object before an infant look began, since an infant look is more likely to have taken place in longer utterances than shorter utterances and vice versa. Note that while the offset can be negative or positive, we included only the absolute value of the offset measure in the model. Finally, we calculated the duration of infants' SA toward an object, as the amount of time infants spent looking at an object after the onset of parent speech.

### 2.7 Data Analysis

Play sessions were on average 6:07 min long (SD = 49 s, range: 4:11 to 7:45). Cumulatively, there were 3517 instances of infant gaze and 3395 coded parent utterances. Together, these resulted in 2275 unique infant looks starting after a parent utterance and 2803 unique parent utterances starting after an infant look. Excluding infant gazes toward parents' faces resulted in 2210 instances of infant‐led interactions (infant gaze to an object followed by parent speech) and 1100 instances of parent‐led interactions (parent speech followed by infant gaze to an object). As parents' utterances could refer to several objects during an infant gaze, the resulting set of infant‐led interactions consisted of 2818 instances. Summary statistics of the main measures can be found in Table 4.

**TABLE 4:** Summary statistics of the study's main measures.

	Total	Infant‐led	Parent‐led
	*M* (range), SD	*M* (range), SD	*M* (range), SD
Infant gaze duration (ms)	6484.877 (1–60263), 8279.046	8187.486 (129–60263), 9272.701	2962.151 (1–31490), 3751.517
Offset duration (ms)	2975.29 (0.818–54131.067), 4856.27	3993.491 (3.888–54131.067), 5614.554	868.616 (0.818–6886.882), 760.798
Speech content (# of occurrences)			
Referential	40.11 (4–110), 26.38	25.52 (2–87), 17.45	14.60 (1–47), 10.59
Social	9.61 (0–31), 7.30	6.10 (0–21), 5.13	3.52 (0–12), 3.09
Situational	22.60 (0–68), 17.06	15.77 (0–55), 12.50	6.82 (0–35), 6.53
Object information	26.69 (1–87), 20.91	17.10 (0–68), 13.97	9.60 (0–36), 8.71
Communicative intent (# of occurrences)			
Declaratives	26.29 (0–89), 19.51	17.23 (0–69), 12.85	9.07 (0–39), 8.07
Imperatives	4.92 (0–22), 4.61	3.48 (0–13), 3.08	1.44 (0–11), 2.26
Descriptive questions	4.27 (0–21), 5.02	2.73 (0–12), 3.16	1.55 (0–12), 2.55
Directive questions	1.24 (0–14), 2.15	0.81 (0–8), 1.32	0.44 (0–6), 1.13
Informative questions	9.15 (0–56), 9.67	6.32 (0–41), 6.82	2.82 (0–15), 3.41
Pedagogical questions	5.39 (0–23), 5.13	3.69 (0–14), 3.47	1.69 (0–12), 2.35

Abbreviations: *M* = mean, SD = standard deviation.

All main models included *age*, the *offset* term and *novelty* as predictors as well as random effects for *participant* and *object*. Additionally, some models included random slopes for dummy coded variables for *alignment* and *novelty*. While running the models, correlations and random slopes were removed if they were one or NA. We *z*‐transformed the *offset* and *age* to aid in model convergence and to ease interpretation of the results. In models with higher order interactions, we also ran reduced models to disentangle the effects. Preliminary versions of all models included the interaction between *alignment* and *novelty* as well as *alignment*, *novelty* and the speech characteristics (see Supporting Information S1: Tables S15–S19 for model output). However, since none of these models found significant interactions between *alignment* and any of the variables, we report only models including *alignment* as a main effect to ease interpretation of other interactions. The syntax for each model can be found in Table 1 (Quinn and Keough 2002).

For models 1.1, 1.2 and 1.3, the response variable (*leader*) was the binary outcome of whether an instance was infant‐led (i.e., infant gaze onset before parent speech onset) or parent‐led (i.e., parent speech onset before infant gaze onset). Infant‐led interactions were coded as 1 and parent‐led interactions as 0. We used generalized linear mixed models (GLMMs) to examine the temporal dynamics of infant gaze and parent speech. Models were specified as binomial models with a logit link function. In model 1.1, we examined the number of caregiver‐child interactions that were infant‐led or parent‐led. We considered all instances of speech and gaze regardless of topical alignment. Model 1.2 expanded on this initial model by including the topical alignment of infant gaze and parent speech as well as the utterance coding on the content level (across four binary variables (1/0) coding for the content of parent speech in each utterance). We coded these as separate variables since each utterance could include instances of two different kinds of speech content. Model 1.3 similarly analyzed the communicative intent of parent utterances (*intent*) across the two types of interactions.

For models 2.1, 2.2 and 2.3, the response variable was the continuous value of *SA*, operationalized as the duration of gaze after the onset of speech. Model 2.1 examined the extent to which *alignment* predicted the duration of SA. Model 2.2 extended this to consider the influence of the content of parent speech on infants' SA. Model 2.3 analyzed the influence of the communicative intent of parent utterances (*intent*) on infant SA.

These models were run separately to improve interpretability of the different factors. To counteract the risk of overinterpretation due to repeated testing of the same outcome variables, we also fit omnibus models for leader‐follower dynamics and infant sustained attention, respectively, including all predictors described above. We conducted full‐null model comparisons to assess whether the predictor set jointly improved model fit relative to a null model. All full–null model comparisons were significant (*Χ* ^2^ = 997.3, *p* < 0.001 for leader‐follower dynamics and *Χ* ^2^ = 78.5, *p* < 0.001 for SA). Full and null model syntaxes and outputs are reported in the Supporting Information S1: Tables S20 and S21.

We ran collinearity and model complexity calculations for all models to ensure the robustness of the effects. Collinearity was assessed using Variance Inflation Factors (VIFs) which were well within the acceptable range (Field 2005; Quinn and Keough 2002). We calculated model complexity to ensure that the dataset was adequate to run the models. The lowest model complexity was 91, establishing that the dataset was adequate to support our models. In addition, we also determined model stability by excluding participants one at a time and fitting the model to each of the derived subsets, and comparing the range of the estimates with those obtained for the full dataset. This revealed the models to be of reasonable stability. We now report these tables in the Supporting Information S1.

All statistical analyses were conducted using R Statistical Software v4.4.3 (R Core Team 2025). We used the packages tidyverse 2.0.0 (Wickham et al. 2019), data.table 1.17.6 (Barrett et al. 2025), and readxl 1.4.5 (Wickham and Bryan 2025) for data processing, and the packages lme4 1.1‐37 (Bates et al. 2015) and psy811 1.0 (Mirman 2019) to fit models and extract *p*‐values.

## 3 Results

### 3.1 Infant‐Led and Parent‐Led Interactions

#### 3.1.1 Differences in the Extent to Which Caregiver‐Child Interactions Are Led by Infant Gaze or Parent Speech (Model 1.1)^3^

**FIGURE 1:**
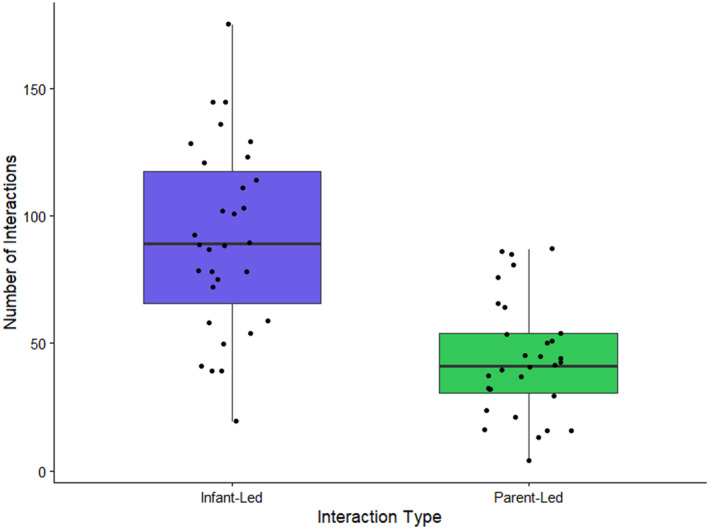
The number of interactions initiated by infant gaze (infant‐led) and parent speech (parent‐led). The horizontal black lines mark the median and quartiles of the response variable. The points represent the raw data. The whiskers extend to the most extreme values within 1.5 IQR (interquartile range) value.

The intercept was significantly positive (Table 5). Thus, overall, more interactions were led by infant gaze than parent speech (Figure 1). There was no significant effect of *novelty*. There was a significant positive effect of the *offset* term: as the time difference between infant gaze and parent speech increases, the more likely it is that the interaction is led by infant gaze than parent speech.

**TABLE 5:** *Output of Model 1.1:* Differences in the extent to which caregiver‐child interactions are led by infant gaze or parent speech.

Predictors	Estimate	SE	95% CI	*z*‐statistic	*p*
Intercept	1.768	0.100	1.572 to 1.964	17.682	**<** **0.001**
Novelty (familiar)	−0.065	0.083	−0.227 to 0.098	−0.777	0.437
Offset^a^	3.703	0.208	3.296 to 4.110	17.839	**<** **0.001**
Age^a^	−0.013	0.041	−0.095 to 0.068	−0.322	0.747

*Note:* Bold values indicate *p* < 0.05.

Abbreviations: CI = confidence interval; SE = standard error.

^a^ *z*‐transformed.

#### 3.1.2 Differences in the Alignment, Novelty and Content of Parent Speech in Infant‐Led and Parent‐Led Interactions (Model 1.2)

**FIGURE 2:**
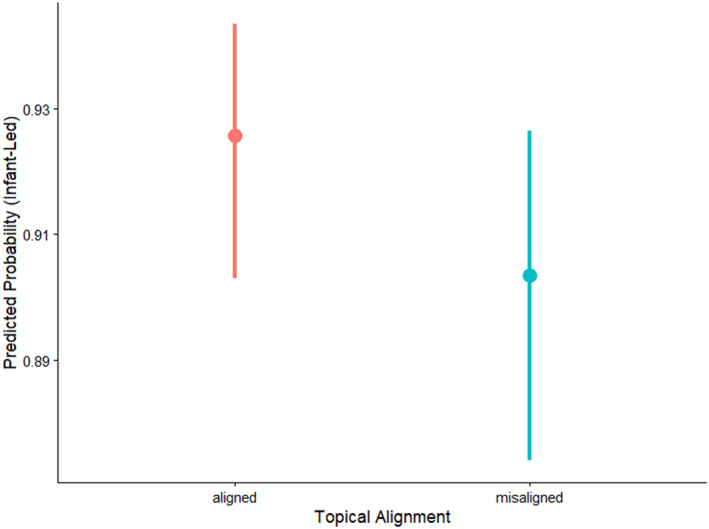
Model predicted probabilities of infant leading as a function of topical alignment. A value of 1 on the *y*‐axis represents a 100% predicted probability of an interaction to be infant‐led, whereas a value of 0 represents a 100% predicted probability of an interaction to be parent‐led (i.e., 0% predicted probability of an interaction to be infant‐led). Thus, the lower the value on the *y*‐axis, the higher the probability of an interaction being parent‐led. The error bars represent 95% confidence intervals.

We found a significant negative effect of *alignment* (Table 6). Infant‐led interactions were more likely to be topically aligned relative to parent‐led interactions (Figure 2). Parent‐led interactions were equally likely to be topically aligned and misaligned. The negative main effect of *referential* speech suggests that parent‐led interactions contained more referential speech relative to infant‐led interactions.

**TABLE 6:** *Output of Model 1.2:* Differences in the alignment, novelty and content of parent speech in infant‐led and parent‐led interactions.

Predictors	Estimate	SE	95% CI	*z*‐statistic	*p*
Intercept	2.524	0.150	2.230 to 2.818	16.831	**<** **0.001**
Alignment (misaligned)	−0.288	0.078	−0.441 to −0.135	−3.681	**<** **0.001**
Offset^a^	3.677	0.186	3.312 to 4.042	19.734	**<** **0.001**
Age^a^	−0.034	0.040	−0.111 to 0.044	−0.846	0.397
Novelty (familiar)	−0.361	0.171	−0.696 to −0.026	−2.115	**0.034**
Referential	−0.455	0.122	−0.695 to −0.215	−3.717	**<** **0.001**
Social	−0.600	0.157	−0.909 to −0.292	−3.816	**<** **0.001**
Situational	−0.181	0.136	−0.447 to 0.086	−1.329	0.184
Object information	−0.375	0.126	−0.623 to −0.128	−2.971	**0.003**
Novelty × referential	0.088	0.167	−0.238 to 0.415	0.529	0.597
Novelty × social	0.760	0.215	0.338 to 1.181	3.533	**<** **0.001**
Novelty × situational	0.141	0.182	−0.215 to 0.496	0.775	0.439
Novelty × object information	0.343	0.173	0.004 to 0.682	1.984	**0.047**

*Note:* Bold values indicate *p* < 0.05.

Abbreviations: CI = confidence interval; SE = standard error.

^a^ *z*‐transformed.

**FIGURE 3:**
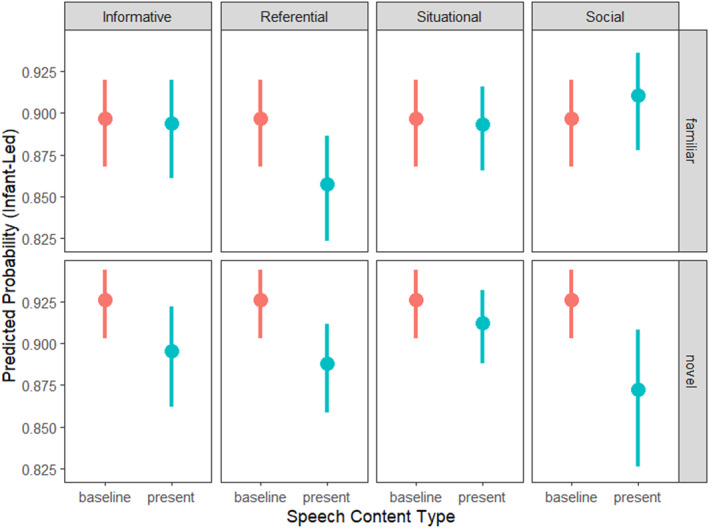
Model predicted probabilities of infant leading as a function of speech content types and object novelty. A value of 1 on the *y*‐axis represents a 100% predicted probability of an interaction to be infant‐led, whereas a value of 0 represents a 100% predicted probability of an interaction to be parent‐led (i.e., 0% predicted probability of an interaction to be infant‐led). Thus, the lower the value on the *y*‐axis, the higher the probability of an interaction being parent‐led. Baseline depicts the predicted probability of an interaction to be infant‐led in the absence of any speech content type, present depicts the predicted probability of an interaction to be infant‐led in the presence of the respective speech type. The error bars represent 95% confidence intervals.

Reduced models breaking down the significant interaction between *novelty* and the content of parent speech showed that, for familiar objects, parents were more likely to use *referential* speech in parent‐led interactions relative to infant‐led interactions (*β* = −0.373, SE = 0.113, *z*‐value = −3.291, *p* = 0.001, see Supporting Information S1: Table S1 for model output). For novel objects, parents are more likely to use *referential* (*β* = −0.458, SE = 0.123, *z*‐value = −3.716, *p* < 0.001), *social* (*β* = −0.598, SE = 0.158, *z*‐value = −3.786, *p* < 0.001) and speech containing *object information* (*β* = −0.377, SE = 0.127, *z*‐value = −2.962, *p* = 0.003, see Supporting Information S1: Table S2 for model output) in parent‐led interactions relative to infant‐led interactions. These findings are illustrated in Figure 3.

#### 3.1.3 Differences in the Alignment, Novelty and Communicative Intent of Parent Speech in Infant‐Led and Parent‐Led Interactions (Model 1.3)

Model 1.3 (Table 7) yielded significant differences in the communicative intent of parent speech concerning novel and familiar objects in infant‐led and parent‐led interactions. While post‐hoc reduced models breaking down the interaction between novelty and communicative intent of parent speech suggested differences between familiar and novel objects (see Supporting Information S1: Tables S3 and S4), we note that these effects were not significant in the omnibus model (see Supporting Information S1: Tables S20 and S21). We, therefore, do not present these results here and treat them with caution.

**TABLE 7:** *Output of Model 1.3*: Differences in the alignment, novelty and communicative intent of parent speech in infant‐led and parent‐led interactions.

Predictors	Estimates	SE	95% CI	*z*‐statistic	*p*
Intercept	2.189	0.148	1.899 to 2.478	14.806	**<** **0.001**
Alignment (misaligned)	−0.285	0.080	−0.442 to −0.127	−3.545	**<** **0.001**
Novelty (familiar)	−0.228	0.161	−0.544 to 0.089	−1.411	0.158
Age^a^	−0.022	0.047	−0.114 to 0.070	−0.475	0.634
Offset^a^	3.669	0.186	3.305 to 4.033	19.762	**<** **0.001**
Declarative	−0.296	0.146	−0.582 to −0.011	−2.037	**0.042**
Imperative	−0.069	0.229	−0.518 to 0.379	−0.303	0.762
Descriptive question	−0.843	0.260	−1.353 to −0.333	−3.240	**0.001**
Directive question	−1.105	0.362	−1.814 to −0.395	−3.051	**0.002**
Informative question	−0.141	0.186	−0.505 to 0.223	−0.761	0.447
Pedagogical question	−0.135	0.227	−0.580 to 0.309	−0.596	0.551
Novelty × declarative	0.105	0.195	−0.278 to 0.488	0.539	0.590
Novelty × descriptive question	0.757	0.343	0.085 to 1.430	2.207	**0.027**
Novelty × directive question	2.488	0.681	1.154 to 3.823	3.655	**<** **0.001**
Novelty × imperative	0.381	0.318	−0.242 to 1.004	1.199	0.230
Novelty × informative question	0.351	0.255	−0.150 to 0.852	1.374	0.169
Novelty × pedagogical question	−0.069	0.311	−0.679 to 0.541	−0.222	0.824

*Note:* Bold values indicate *p* < 0.05.

Abbreviations: CI = confidence interval; SE = standard error.

^a^ *z*‐transformed.

### 3.2 Sustained Attention

#### 3.2.1 The Role of Topical Alignment of Parent Speech in Sustaining Infant Gaze (Model 2.1)

**FIGURE 4:**
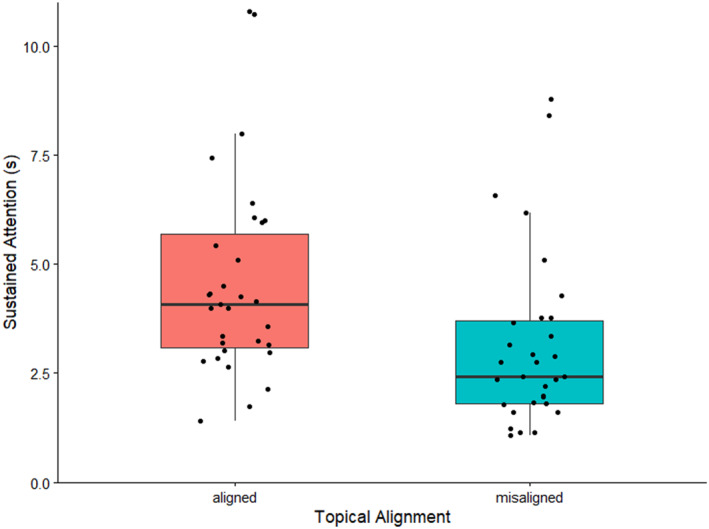
The distribution of sustained attention in seconds across topical alignment. The horizontal black lines mark the median and quartiles of the response variable. The points represent the raw data. The whiskers extend to the most extreme values within 1.5 IQR.

The model (Table 8) yielded a significant positive intercept, that is, SA is increased for a novel object in a topically aligned interaction. Furthermore, we found a significant negative effect of *alignment*, suggesting that infants show significantly less SA to an object when parent speech is topically misaligned to this object (Figure 4). The *offset* term was significant and positive suggesting that the longer infants have attended to an object before parents begin talking, the more likely it is for infants to sustain attention to this object for longer.

**TABLE 8:** *Output of Model 2.1:* The role of topical alignment of parent speech in sustaining infant gaze.

Predictors	Estimates	SE	95% CI	*t*‐statistic	*p*
Intercept	4595.228	562.995	3491.300 to 5699.155	8.162	**<** **0.001**
Alignment (misaligned)	−1240.538	397.856	−2020.658 to −460.418	−3.118	**0.002**
Novelty (familiar)	−93.021	552.297	−1175.970 to 989.928	−0.168	0.866
Offset^a^	752.123	112.951	530.648 to 973.599	6.659	**<** **0.001**
Age^a^	10.581	281.804	−541.984 to 563.146	0.038	0.970

*Note:* Bold values indicate *p* < 0.05.

Abbreviations: CI = confidence interval; SE = standard error.

^a^ *z*‐transformed.

#### 3.2.2 Differences in the Alignment, Novelty and Content of Parent Speech in Sustaining Infant Gaze (Model 2.2)

The model yielded a significant positive intercept, a significant negative effect of *alignment* and a significant positive effect of the *offset* term (Table 9). In addition, we found a significant positive effect of the interaction between *novelty* and *situational* speech and *novelty* and speech containing *object information* on sustaining infant gaze. Thus, relative to novel objects, for a familiar object, SA increases when parents use *situational* speech or speech containing *object information*. We note, however, that the interaction between *novelty* and *situational* speech was not significant in the omnibus model.

**TABLE 9:** *Output of Model 2.2:* The role of alignment, novelty and content of parent speech in sustaining infant gaze.

Predictors	Estimate	SE	95% CI	*t*‐statistic	*p*
Intercept	4799.338	629.591	3564.827 to 6033.848	7.623	**<** **0.001**
Alignment (misaligned)	−1243.031	403.464	−2034.149 to −451.913	−3.081	**0.002**
Offset^a^	743.968	113.340	521.729 to 966.208	6.564	**<** **0.001**
Age^a^	−14.834	280.232	−564.316 to 534.648	−0.053	0.958
Novelty (familiar)	−692.684	664.877	−1996.383 to 611.014	−1.042	0.298
Referential	268.598	343.463	−404.869 to 942.064	0.782	0.434
Social	218.102	494.407	−751.338 to 1187.543	0.441	0.659
Situational	−488.964	374.223	−1222.747 to 244.818	−1.307	0.191
Object information	−639.655	372.716	−1370.482 to 91.173	−1.716	0.086
Novelty × referential	−523.212	452.607	−1410.690 to 364.265	−1.156	0.248
Novelty × social	−708.945	640.613	−1965.068 to 547.178	−1.107	0.269
Novelty × situational	1113.156	495.489	141.596 to 2084.717	2.247	**0.025**
Novelty × object information	1682.014	495.712	710.015 to 2654.013	3.393	**0.001**

*Note:* Bold values indicate *p* < 0.05.

Abbreviations: CI = confidence interval; SE = standard error.

^a^ *z*‐transformed.

Reduced models suggest that, for familiar objects, speech containing *object information* sustains infants' attention to an object for longer (*β* = 1206.871, SE = 347.295, *t*‐value = 3.475, *p* = 0.001, see Supporting Information S1: Table S5). In contrast, there was no main effect of speech content on infants' SA to novel objects (all *p* ≥ 0.147, see Supporting Information S1: Table S6).

#### 3.2.3 Differences in the Alignment, Novelty and Communicative Intent of Parent Speech in Sustaining Infant Gaze (Model 2.3)

The model (Table 10) yielded a significant positive intercept, a significant negative effect of *alignment* and a significant positive effect of the *offset* term (as in Model 2 above). In addition, we found a significant negative effect of the interaction between *novelty* and *directive questions* and *novelty* and *informative questions* on sustaining infant gaze. Thus, relative to novel objects, for familiar objects, SA decreases when parents use directive or informative questions.

**TABLE 10:** *Output of Model 2.3:* The role of alignment, novelty and communicative intent of parent speech in sustaining infant gaze.

Predictors	Estimates	SE	95% CI	*t*‐statistic	*p*
Intercept	4257.575	630.779	3020.734 to 5494.417	6.750	**<** **0.001**
Alignment (misaligned)	−1270.691	395.279	−2045.760 to −495.622	−3.215	**0.001**
Novelty (familiar)	344.941	669.717	−968.250 to 1658.133	0.515	0.607
Age^a^	17.088	282.645	−537.127 to 571.302	0.060	0.952
Offset^a^	768.596	113.066	546.896 to 990.296	6.798	**<** **0.001**
Declarative	94.946	411.623	−712.171 to 902.062	0.231	0.818
Descriptive question	−359.394	801.293	−1930.581 to 1211.793	−0.449	0.654
Directive question	2888.567	1272.533	393.366 to 5383.768	2.270	**0.023**
Imperative	320.199	631.259	−917.583 to 1557.981	0.507	0.612
Informative question	1629.411	537.772	574.939 to 2683.882	3.030	**0.002**
Pedagogical question	438.494	606.432	−750.606 to 1627.593	0.723	0.470
Novelty × declarative	166.051	548.589	−909.630 to 1241.732	0.303	0.762
Novelty × imperative	−345.926	857.666	−2027.649 to 1335.798	−0.403	0.687
Novelty × descriptive question	−26.012	995.088	−1977.195 to 1925.171	−0.026	0.979
Novelty × directive question	−3944.049	1637.397	−7154.680 to −733.419	−2.409	**0.016**
Novelty × informative question	−2265.786	709.670	−3657.317 to −874.254	−3.193	**0.001**
Novelty × pedagogical question	−892.509	854.748	−2568.511 to 783.493	−1.044	0.296

*Note:* Bold values indicate *p* < 0.05.

Abbreviations: CI = confidence interval; SE = standard error.

^a^ *z*‐transformed.

**FIGURE 5:**
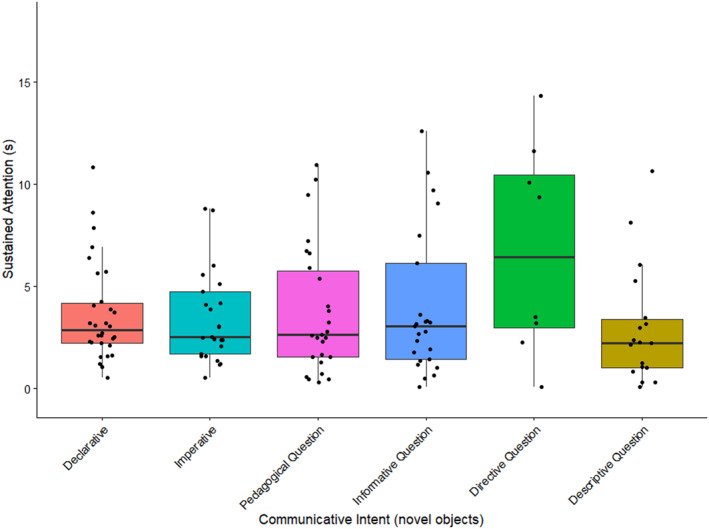
The distribution of sustained attention in seconds across different communicative intent types for novel objects. The horizontal black lines mark the median and quartiles of the response variable. The points represent the raw data. The whiskers extend to the most extreme values within 1.5 IQR. Two extreme values above 30 s for *Imperative* and *Informative Question* have been removed for better readability. The full figure including all data points can be found in Supporting Information S1: Figure S1.

Indeed, reduced models suggest that, for familiar objects, there was no main effect of different communicative intent types on infants' SA (all *p* ≥ 0.133, see Supporting Information S1: Table S7). In contrast, *directive* (*β* = 2808.035, SE = 1151.295, *t*‐value = 2.439, *p* = 0.015) and *informative* (*β* = 1615.204, SE = 488.580, *t*‐value = 3.306, *p* = 0.001) questions sustained infants' attention to novel objects for longer, albeit regardless of whether parents were talking about the object or not (see Supporting Information S1: Table S8). These findings are illustrated in Figure 5.

## 4 Discussion

The present study examined the role of parents' speech with regard to two aspects of caregiver‐child interactions during free play: (1) whether interactions were more likely to be led by infant gaze or parent speech, and (2) the extent to which infants' SA to an object is associated with specific characteristics of parent speech. In what follows, we discuss the results of the study with regard to each of the above points.

### 4.1 Speech in Infant‐Led and Parent‐Led Interactions

Across all models comparing infant‐led and parent‐led interactions, interactions were more likely to be initiated by an infant look toward an object than parents' speech about an object. Infant‐led interactions were also more likely to be topically aligned than parent‐led interactions, that is, parents were more likely to talk about the object that the infant was looking at, whereas infants were less likely to look at the object that their parent was talking about. Interactions also were also more likely to be infant‐led the longer infants had gazed at the object prior to speech onset.

These results align with previous studies showing that, in the second year of life, infants commonly lead the focus of attention in free play interactions, whereas parents join their infants' focus of attention and provide relevant information responsively (Chang et al. 2016; Goupil et al. 2024; Madhavan et al. 2026). While previous studies focused on the extent to which interactions may be led by infant gaze as opposed to parent gaze or parent touch, the current study investigated whether parent speech may be associated with the focus of attention in caregiver‐child interactions. Contrary to our expectations, but extending previous findings, we find that caregiver‐child interactions are more likely to be led by infant gaze than *even* by parent speech. This was especially the case when infants had looked at an object for an extended time prior to parent speech. Thus, when parents responded to their infant's extended looks toward an object by talking about it, they may have provided information about this object when their infants were especially receptive to it. Additionally, infants' extended gaze toward an object may have made it easier for parents to realize what their infants were focusing on, enabling them to respond more contingently.

We found that parent‐led interactions were less likely to be topically aligned than infant‐led interactions, that is, infants' gaze shifts were less likely to be toward the object the parent was talking about. This resonates with previous evidence that infants were less likely to join their parents' focus of attention when they shifted their gaze after parents' speech onset (Goupil et al. 2024). Similarly, C. Yu et al. (2021) found that, while 19‐month‐old infants' gaze after a parent's object naming is often focused on one object, this object is equally likely to be the correct or the incorrect referent of the utterance. The authors argue that this is likely due to the complexity of multimodal interactions between infants and caregivers during free play situations, and that referential ambiguity (from the infants' perspective) cannot be solved by one single cue, but only by the interplay of all available cues in an interaction. In keeping with this, Deák et al. (2008) found that pointing is as effective at redirecting infants' gaze toward a target object as using speech, and that 21‐month‐old infants follow both re‐directive utterances and points more often than 15‐month‐olds, indicating that infants become better at following their caregivers' directive cues with increasing age. While the present study did not investigate parents' multimodal communication, using the same dataset, Madhavan et al. (2026) report that parents were more likely to manipulate the focused objects when they were leading the instance of joint attention. Thus, in this dataset, parent touch may have provided infants with a consistent cue as to what parents are talking about. Future studies could, therefore, examine the combined effects of parents' speech, gaze, and object manipulation to further elucidate the dynamic, multimodal process of establishing joint attention between caregivers and their infants.

Finally, we did not find significant overall effects of novelty on whether an interaction was more likely to be infant‐led or parent‐led. However, as we discuss below, the characteristics of parent speech differ considerably across interactions focusing on novel and familiar objects, thereby outlining the mechanisms underlying previously reported effects of novelty on caregiver‐child interactions (Chen et al. 2021; Goupil et al. 2024; Madhavan et al. 2026).

### 4.2 Speech and Sustained Attention

Analyses examining the interaction between parent speech and infant gaze duration further clarified parents' role in caregiver‐child interactions. Infant‐led parent speech was more likely to be associated with infants' sustained attention to an object when it was topically aligned to the infant gaze, especially when infants had already been attending to the object for an extended amount of time. Parents' misaligned speech was associated with infants' shortened gaze toward their original focus of attention. Thus, the positive relationship between parent speech and infant SA is contingent on the speech being aligned with infant attention.

Several studies have shown that parents' speech is associated with infants' SA for an object (Schroer et al. 2019; Suarez‐Rivera et al. 2019), indicating that speech scaffolds 1–2 year olds' SA. However, these studies have either not considered the topical alignment between parent speech and infant gaze (Schroer et al. 2019), or only considered topically aligned utterances (Suarez‐Rivera et al. 2019). Our findings indicate that parent speech was strongly associated with infants' SA when it is topically aligned with the infants' focus of attention. Thus, it does not seem to be speech alone that serves to sustain infants' attention to an object—rather, the topical alignment of speech to infants' gaze is associated with infants' engagement with an object.

Furthermore, the interaction between parent speech and infants' SA was positively moderated by infant gaze duration before speech onset. Infant gaze duration prior to speech may, therefore, provide a fine‐grained index of infants' “readiness to learn” and the positive effect of temporally and semantically aligned input at such moments in time. Longer looking to an object prior to parent speech may also have been accompanied by infant verbal or multimodal bids for information, leading to parents being able to provide information relevant to infants' visual focus. An interesting avenue for future research would, therefore, be to consider the multimodality of infants' bids for information and their impact on subsequent attention and encoding.

### 4.3 Linguistic Characteristics of Parent Speech

A particular focus of the present study was on examining the linguistic characteristics of parent speech across the variety of interactions described above, in an attempt to disentangle their role in caregiver‐child interactions. One overarching finding was that, when initiating an interaction, parents used mostly referential speech, that is, speech referring to an object using labels, pronouns, or noises. This applied regardless of the topical alignment of parent speech and infant gaze. Thus, referential speech appears to be related to infants' shifts of attention away from one object toward another, but not necessarily to the object that is being talked about. This finding did not differ across novel and familiar objects, indicating that infants' missing understanding of novel object labels was likely not the underlying cause of this pattern. Furthermore, referential speech did not predict infants' SA toward an object, suggesting that referential speech may not be mainly associated with infants' SA but rather with infants' shifts of attention in caregiver‐child interactions, albeit not necessarily to the intended object.

Indeed, we found no interaction between topical alignment and any kind of speech that parents directed toward infants. Parent speech neither differed when infants and parents were attending to the same or to different objects, nor were different speech types more or less likely to be associated with shifts in infants' attention toward any objects. This speaks to the limited malleability of infants' attention through speech in early interactions, corroborating studies highlighting the egocentricity of infants in caregiver‐child interactions (Franchak et al. 2018; Madhavan et al. 2026). As mentioned above, multimodal communication may make the difference between successful and unsuccessful redirection, where parents' pointing or manipulation of objects may give infants the necessary clarification of the correct referent. Future studies might test this idea by investigating parents' multimodal communication and comparing the success of parent utterances with and without concurrent manual activities at redirecting infant attention.

The increased use of referential speech in parent‐led interactions was common to interactions concerning novel and familiar objects. As all other relevant speech types differed substantially across such interactions, the remaining results will be discussed separately for novel and familiar objects in the following sections.

#### 4.3.1 Familiar Objects

Parent speech about familiar objects in the play setting appeared to be mostly object‐focused: parent‐led interactions were more likely to include referential speech than infant‐led interactions, and infants' extended SA to familiar objects was associated with speech containing object information. This matches findings by Peters and Yu (2020) that speech containing object information was most associated with infants' SA. Taken together with Madhavan et al. (2026)'s findings that infants are more likely to initiate interactions with familiar relative to novel objects, it is possible that familiar objects provide dyads with a “common ground” of knowledge (L. Goupil, personal communication, June 27, 2025), allowing parents to focus their speech on the characteristics and actions related to the objects. Thus, when parents initiate an interaction with a familiar object, they may rely on their shared familiarity with the object, and the infants' inherent interest in familiar objects to draw and sustain infants' attention to the object.

Some models indicated that infant‐led interactions were more likely to be characterized by directive questions, and that infant SA was associated with situational speech. However, since these results were not consistent across the omnibus and split models, we refrain from further interpretation.

#### 4.3.2 Novel Objects

Parents' speech in interactions focusing on novel objects was characterized by a greater variety of speech compared to familiar objects. Relative to infant‐led interactions, in parent‐led interactions concerning novel objects, parents used more referential, social, and object information speech. We interpret these findings as indicating that parents used more varied speech when talking about a novel object, perhaps in an attempt to catch infants' attention and find “common ground”. Furthermore, in infant‐led interactions, infants' attention to a novel object was extended when parents used informative and directive questions. These were commonly related to the infants' mental state, and it is possible that parents felt the need to ask their infants about how they felt and what they wanted to do with an object, which may have prolonged infants' attention to these objects. As with the familiar objects, we note that some models indicated that, compared to infant‐led interactions, parent‐led interactions contained more declaratives, descriptive questions, and directive questions. However, we refrain from further interpreting these effects given that they were not consistent across models.

Overall, our findings suggest that parent speech is influenced by infants' familiarity with an object, with parents providing greater variety in their speech about novel objects relative to familiar objects. We tentatively interpret these findings as suggesting that parents may have included greater variety in their speech regarding novel objects in an attempt to direct and sustain their child's attention on these objects, while mostly referring to familiar objects in play with their child. This may be related to the “common ground” of shared knowledge about familiar objects (e.g., familiar routines and play) that may be immediately available in interactions concerning familiar objects, while parents may be attempting to establish such routines in interactions concerning novel objects. This could be further investigated by analyzing the repetitiveness of play actions and caregiver utterances in caregiver‐child‐interactions with familiar compared to novel objects. If it is, indeed, the case that familiar objects provide an opportunity to follow play patterns that are typical to these objects and familiar to both partners, it is likely that these would be repeated more often than play actions with novel objects, where patterns still need to be established.

### 4.4 Limitations

The present study has some limitations. First, we note that, given the number of predictors under examination in the current study, we ran several models with the same outcome variable, which means that the results are subject to the problem of multiple testing. To limit this problem, we ran a full‐null comparison on an omnibus model including all predictor variables which showed a very similar pattern of results (see Supporting Information S1: Tables S20–S25). Consequently, we only discuss results that were consistent across the omnibus and individual models.

We also note that the final sample included in the analysis has a relatively wide age range—from 14 to 23 months. Over the second year of life, infants show enormous developmental progress on many different levels, such as their motor, language, and communicative abilities. While we found no significant effects of age in any of our analyses, it is possible that this is due to a lack of statistical power. Additionally, the sample contained an uneven distribution of parent gender, with a much larger number of mothers than fathers. Although previous studies have found differences in how mothers and fathers interact with their infants, removing fathers from our analyses did not change the statistical results. Increasing the sample size and balancing parent gender in future studies would provide better insights into the existence of such effects.

The current study also focused only on the interaction of infants' gaze and parents' utterances. As mentioned above, both infants and parents likely use a much wider range of modalities when interacting, and ultimately, caregiver‐child interactions can only be better understood from a multimodal perspective. We note that the multimodality of parent‐child interactions was the focus of an earlier study examining this dataset and refer the reader to this study (Madhavan et al. 2026). Here, we found only that parents were more likely to touch an object that they were talking about, while finding no influence of parent touch on infant attention.

Finally, the speech type categories that characterized parent‐led interactions—especially referential and object information speech—are quite broad and could contain many different types of utterances about an object. More detailed analyses of the exact speech content in these speech types might elucidate further whether there are specific types of information that attract and sustain infants' attention more than others.

## 5 Conclusion

In conclusion, the present study examined the temporal dynamics of different parent speech characteristics and infant attention in caregiver‐child interactions. We found that caregiver‐child interactions were overwhelmingly more likely to be led by infant gaze as opposed to parent speech. We expanded previous findings by showing that parents follow their infants' attention—not just with their gaze—but also their speech, both in the temporal dimension and with regards to the topic of interest, and that this readiness to follow is associated with infants' extended attention for both novel and familiar objects. Furthermore, we showed that parents' speech differs between interactions with novel and familiar objects, especially when parent speech preceded infants' gaze shifts. Speech about familiar objects was mostly object‐focused, whereas speech about novel objects was more varied, referring not only to an object, but also referencing the partners in the dyad. We interpret this pattern as suggesting that familiar objects provide “common ground” between the infants and parents, which structures interactions with and parents' speech about the objects and engages infants more easily, while novel objects lack such a shared knowledge for interactions, thereby potentially forcing parents to include greater variability in their speech.

## Author Contributions

**Anne‐Kathrin Mahlke:** conceptualization, data curation, investigation, methodology, project administration, writing – original draft, writing – review and editing, software, visualization, formal analysis. **Shreya Venkatesan:** data curation, formal analysis, methodology, software, visualization, writing – original draft. **Nivedita Mani:** conceptualization, data curation, formal analysis, funding acquisition, methodology, resources, supervision, writing – original draft, writing – review and editing, visualization.

## Ethics Statement

The present study adhered to the principles outlined in the Declaration of Helsinki. Before any recording or data collection, parents provided informed consent for their and their infants' participation. Approval for all procedures involving human subjects was received from the ethics committee of the Georg‐Elias‐Müller Institute for Psychology, Georg August University of Göttingen. Each child received a children's book as a token of appreciation for their participation.

## Conflicts of Interest

The authors declare no conflicts of interest.

## Supporting information


Supporting Information S1

## Data Availability

Anonymized raw data files and the R script used for analysis are openly available at the project's Open Science Framework page (https://osf.io/f2qwz/).

## Endnotes

^1^
No names were provided to the parents, so names for some toys differed between dyads.

^2^
An overview of the C‐unit segmentation rules can be found here: https://www.saltsoftware.com/resources/tranaids.

^3^
Since the sample included more mothers than fathers, we reran all models excluding the data from the father‐child interactions. All patterns presented here equally emerged for mother‐only data and can be found in the (Supporting Information S1: Tables S9–S14).
