# Supplementary material for: Parent Speech in Free Play Is Guided by Infant Attention, But Organized by Object Familiarity
Source: Infancy. 2026 May 4;31:e70089. doi: 10.1111/infa.70089 (PMC13139530; doi:10.1111/infa.70089)
Supplement: Supplementary file 1 — Supporting Information S1 [file INFA-31-0-s001.docx]

**Supplementary Information**

**Parent Speech in Free Play Is Guided by Infant Attention, but Organised by Object Familiarity**

**GLMM Results**

**Table S1**

*Output of Model 1.2a:* Differences in the alignment, novelty and content of parent speech in infant-led and parent-led interactions with **familiar objects**

| *Predictors* | *Log-Odds* | *SE* | *95% CI* | *Statistic* | *p* |
| --- | --- | --- | --- | --- | --- |
| Intercept | 2.426 | 0.173 | 2.087 – 2.765 | 14.029 | **<.001** |
| Alignment (misaligned) | -0.382 | 0.103 | -0.583 – -0.180 | -3.708 | **<.001** |
| Offset^1^ | 3.865 | 0.266 | 3.345 – 4.385 | 14.557 | **<.001** |
| Age^1^ | -0.049 | 0.051 | -0.149 – 0.051 | -0.958 | .338 |
| Referential | -0.373 | 0.113 | -0.596 – -0.151 | -3.291 | **.001** |
| Social | 0.161 | 0.146 | -0.125 – 0.448 | 1.103 | .270 |
| Situational | -0.038 | 0.120 | -0.273 – 0.198 | -0.314 | .753 |
| Object Information | -0.022 | 0.119 | -0.255 – 0.212 | -0.181 | .856 |

*Note.* *SE* = standard error; CI = confidence interval. ^1^z-transformed

**Table S2**

*Output of Model 1.2b:* Differences in the alignment, novelty and content of parent speech in infant-led and parent-led interactions with **novel objects**

| *Predictors* | *Log-Odds* | *SE* | *95% CI* | *Statistic* | *p* |
| --- | --- | --- | --- | --- | --- |
| Intercept | 2.201 | 0.165 | 1.878 – 2.524 | 13.353 | **<.001** |
| Alignment (misaligned) | -0.185 | 0.110 | -0.400 – 0.031 | -1.677 | .094 |
| Offset^1^ | 3.403 | 0.256 | 2.902 – 3.904 | 13.308 | **<.001** |
| Age^1^ | -0.014 | 0.059 | -0.131 – 0.102 | -0.237 | .813 |
| Referential | -0.458 | 0.123 | -0.699 – -0.216 | -3.716 | **<.001** |
| Social | -0.598 | 0.158 | -0.908 – -0.288 | -3.786 | **<.001** |
| Situational | -0.181 | 0.137 | -0.449 – 0.088 | -1.317 | .188 |
| Object Information | -0.377 | 0.127 | -0.627 – -0.128 | -2.962 | **.003** |

*Note.* *SE* = standard error; CI = confidence interval. ^1^z-transformed

**Table S3**

*Output of Model 1.3a:* Differences in the alignment, novelty and communicative intent of parent speech in infant-led and parent-led interactions with **familiar objects**

| *Predictors* | *Log-Odds* | *SE* | *95% CI* | *Statistic* | *p* |
| --- | --- | --- | --- | --- | --- |
| Intercept | 2.224 | 0.162 | 1.907 – 2.542 | 13.735 | **<.001** |
| Alignment (misaligned) | -0.378 | 0.104 | -0.582 – -0.175 | -3.641 | **<.001** |
| Offset^1^ | 3.869 | 0.267 | 3.345 – 4.392 | 14.483 | **<.001** |
| Age^1^ | -0.048 | 0.056 | -0.159 – 0.062 | -0.853 | .394 |
| Declarative | -0.192 | 0.132 | -0.450 – 0.066 | -1.461 | .144 |
| Imperative | 0.304 | 0.221 | -0.129 – 0.737 | 1.375 | .169 |
| Descriptive Question | -0.051 | 0.227 | -0.495 – 0.393 | -0.225 | .822 |
| Directive Question | 1.381 | 0.576 | 0.252 – 2.509 | 2.398 | **.016** |
| Informative Question | 0.219 | 0.177 | -0.128 – 0.566 | 1.237 | .216 |
| Pedagogical Question | -0.210 | 0.214 | -0.630 – 0.210 | -0.980 | .327 |

*Note.* *SE* = standard error; CI = confidence interval. ^1^z-transformed

**Table S4**

*Output of Model 1.3b:* Differences in the alignment, novelty and communicative intent of parent speech in infant-led and parent-led interactions with **novel objects**

| *Predictors* | *Log-Odds* | *SE* | *95% CI* | *Statistic* | *p* |
| --- | --- | --- | --- | --- | --- |
| Intercept | 1.854 | 0.166 | 1.529 – 2.178 | 11.188 | **<.001** |
| Alignment (misaligned) | -0.186 | 0.110 | -0.401 – 0.029 | -1.693 | .090 |
| Offset^1^ | 3.378 | 0.252 | 2.885 – 3.871 | 13.430 | **<.001** |
| Age^1^ | 0.007 | 0.068 | -0.126 – 0.139 | 0.097 | .923 |
| Declarative | -0.293 | 0.147 | -0.582 – -0.005 | -1.992 | **.046** |
| Imperative | -0.065 | 0.230 | -0.517 – 0.386 | -0.284 | .777 |
| Descriptive Question | -0.865 | 0.265 | -1.384 – -0.347 | -3.270 | **.001** |
| Directive Question | -1.152 | 0.367 | -1.872 – -0.432 | -3.138 | **.002** |
| Informative Question | -0.142 | 0.188 | -0.510 – 0.226 | -0.755 | .450 |
| Pedagogical Question | -0.135 | 0.228 | -0.582 – 0.311 | -0.593 | .553 |

*Note.* *SE* = standard error; CI = confidence interval. ^1^z-transformed

**Table S5**

*Output of Model 2.2a:* Differences in the alignment, novelty and content of parent speech in sustaining infant gaze in interactions with **familiar objects**

| *Predictors* | *Estimate* | *SE* | *95% CI* | *Statistic* | *p* |
| --- | --- | --- | --- | --- | --- |
| Intercept | 4163.222 | 1052.752 | 2098.232 – 6228.211 | 3.955 | **<.001** |
| Alignment (misaligned) | -959.978 | 912.126 | -2749.127 – 829.172 | -1.052 | .293 |
| Offset^1^ | 1192.887 | 160.401 | 878.258 – 1507.515 | 7.437 | **<.001** |
| Age^1^ | -414.883 | 211.244 | -829.241 – -0.525 | -1.964 | **.050** |
| Referential | -311.826 | 315.423 | -930.535 – 306.882 | -0.989 | .323 |
| Social | -561.289 | 434.276 | -1413.129 – 290.551 | -1.292 | .196 |
| Situational | 648.025 | 346.189 | -31.031 – 1327.082 | 1.872 | .061 |
| Object Information | 1206.871 | 347.295 | 525.647 – 1888.096 | 3.475 | **.001** |

*Note.* *SE* = standard error; CI = confidence interval. ^1^z-transformed

**Table S6**

*Output of Model 2.2b:* Differences in the alignment, novelty and content of parent speech in sustaining infant gaze in interactions with **novel objects**

| *Predictors* | *Estimate* | *SE* | *95% CI* | *Statistic* | *p* |
| --- | --- | --- | --- | --- | --- |
| Intercept | 4505.291 | 510.844 | 3503.092 – 5507.490 | 8.819 | **<.001** |
| Alignment (misaligned) | -1028.011 | 362.160 | -1738.514 – -317.508 | -2.839 | **.005** |
| Offset^1^ | 288.999 | 152.509 | -10.202 – 588.199 | 1.895 | .058 |
| Age^1^ | -65.400 | 400.674 | -851.462 – 720.661 | -0.163 | .870 |
| Referential | 162.705 | 313.632 | -452.594 – 778.004 | 0.519 | .604 |
| Social | 208.347 | 449.319 | -673.149 – 1089.843 | 0.464 | .643 |
| Situational | -463.109 | 340.071 | -1130.277 – 204.059 | -1.362 | .174 |
| Object Information | -492.334 | 339.309 | -1158.008 – 173.340 | -1.451 | .147 |

*Note.* *SE* = standard error; CI = confidence interval. ^1^z-transformed

**Table S7**

*Output of Model 2.3a:* Differences in the alignment, novelty and communicative intent of parent speech in sustaining infant gaze in interactions with **familiar objects**

| *Predictors* | *Estimate* | *SE* | *95% CI* | *Statistic* | *p* |
| --- | --- | --- | --- | --- | --- |
| Intercept | 9080.912 | 825.291 | 7462.092 – 10699.731 | 11.003 | **<.001** |
| Alignment (misaligned) | -1155.453 | 317.013 | -1777.279 – -533.627 | -3.645 | **<.001** |
| Offset^1^ | 7084.159 | 162.306 | 6765.793 – 7402.525 | 43.647 | **<.001** |
| Age^1^ | 260.889 | 357.244 | -439.850 – 961.627 | 0.730 | .465 |
| Declarative | 268.739 | 387.883 | -492.099 – 1029.578 | 0.693 | .489 |
| Imperative | 80.539 | 623.611 | -1142.683 – 1303.760 | 0.129 | .897 |
| Descriptive Question | -440.418 | 636.814 | -1689.538 – 808.702 | -0.692 | .489 |
| Directive Question | -876.507 | 1106.178 | -3046.292 – 1293.278 | -0.792 | .428 |
| Informative Question | -747.951 | 497.583 | -1723.967 – 228.065 | -1.503 | .133 |
| Pedagogical Question | -289.418 | 644.400 | -1553.417 – 974.581 | -0.449 | .653 |

*Note.* *SE* = standard error; CI = confidence interval. ^1^z-transformed

**Table S8**

*Output of Model 2.3b:* Differences in the alignment, novelty and communicative intent of parent speech in sustaining infant gaze in interactions with **novel objects**

| *Predictors* | *Estimate* | *SE* | *95% CI* | *Statistic* | *p* |
| --- | --- | --- | --- | --- | --- |
| Intercept | 7361.931 | 516.161 | 6349.302 – 8374.560 | 14.263 | **<.001** |
| Alignment (misaligned) | -997.679 | 289.200 | -1565.045 – -430.313 | -3.450 | **.001** |
| Offset^1^ | 5463.892 | 151.739 | 5166.203 – 5761.582 | 36.008 | **<.001** |
| Age^1^ | -114.522 | 405.078 | -909.223 – 680.179 | -0.283 | .777 |
| Declarative | 173.743 | 374.122 | -560.228 – 907.713 | 0.464 | .642 |
| Imperative | 351.358 | 572.694 | -772.180 – 1474.895 | 0.614 | .540 |
| Descriptive Question | -142.389 | 726.646 | -1567.958 – 1283.181 | -0.196 | .845 |
| Directive Question | 2808.035 | 1151.295 | 549.370 – 5066.701 | 2.439 | **.015** |
| Informative Question | 1615.204 | 488.580 | 656.685 – 2573.722 | 3.306 | **.001** |
| Pedagogical Question | 526.883 | 550.657 | -553.421 – 1607.187 | 0.957 | .339 |

*Note.* *SE* = standard error; CI = confidence interval. ^1^z-transformed

**Table S9**

*Model 1.1 Mothers only:* Differences in the extent to which caregiver-child interactions are led by infant gaze or parent speech

| *Predictors* | *Log-Odds* | *SE* | *95% CI* | *Statistic* | *p* |
| --- | --- | --- | --- | --- | --- |
| Intercept | 1.914 | 0.117 | 1.685 – 2.144 | 16.359 | **<.001** |
| Novelty (familiar) | -0.078 | 0.092 | -0.257 – 0.102 | -0.849 | .396 |
| Offset^1^ | 4.036 | 0.247 | 3.552 – 4.520 | 16.353 | **<.001** |
| Age^1^ | 0.028 | 0.046 | -0.063 – 0.118 | 0.598 | .550 |

*Note.* *SE* = standard error; CI = confidence interval. ^1^z-transformed

**Table S10**

*Model 1.2 Mothers only:* Differences in the alignment, novelty and content of parent speech in infant-led and parent-led interactions

| *Predictors* | *Log-Odds* | *SE* | *95% CI* | *Statistic* | *p* |
| --- | --- | --- | --- | --- | --- |
| Intercept | 2.736 | 0.170 | 2.403 – 3.069 | 16.120 | **<.001** |
| Alignment (misaligned) | -0.249 | 0.085 | -0.415 – -0.083 | -2.945 | **.003** |
| Offset^1^ | 4.086 | 0.225 | 3.645 – 4.527 | 18.168 | **<.001** |
| Novelty (familiar) | -0.431 | 0.188 | -0.800 – -0.062 | -2.292 | **.022** |
| Referential | -0.499 | 0.135 | -0.763 – -0.235 | -3.706 | **<.001** |
| Social | -0.716 | 0.177 | -1.063 – -0.370 | -4.048 | **<.001** |
| Situational | -0.110 | 0.147 | -0.398 – 0.178 | -0.749 | .454 |
| Object Information | -0.362 | 0.140 | -0.636 – -0.088 | -2.593 | **.010** |
| Age^1^ | -0.004 | 0.048 | -0.098 – 0.091 | -0.074 | .941 |
| Novelty × Referential | 0.168 | 0.183 | -0.190 – 0.527 | 0.921 | .357 |
| Novelty × Social | 0.908 | 0.241 | 0.436 – 1.379 | 3.770 | **<.001** |
| Novelty × Situational | 0.045 | 0.197 | -0.342 – 0.431 | 0.227 | .820 |
| Novelty × Object Information | 0.351 | 0.192 | -0.025 – 0.727 | 1.831 | .067 |

*Note.* *SE* = standard error; CI = confidence interval. ^1^z-transformed

**Table S11**

*Model 1.3 Mothers only:* Differences in the alignment, novelty and communicative intent of parent speech in infant-led and parent-led interactions

| *Predictors* | *Log-Odds* | *SE* | *95% CI* | *Statistic* | *p* |
| --- | --- | --- | --- | --- | --- |
| Intercept | 2.468 | 0.168 | 2.138 – 2.797 | 14.687 | **<.001** |
| Alignment (misaligned) | -0.249 | 0.087 | -0.419 – -0.078 | -2.860 | **.004** |
| Novelty (familiar) | -0.365 | 0.177 | -0.712 – -0.018 | -2.063 | **.039** |
| Declarative | -0.404 | 0.160 | -0.717 – -0.091 | -2.529 | **.011** |
| Descriptive Question | -0.872 | 0.295 | -1.450 – -0.293 | -2.953 | **.003** |
| Directive Question | -1.088 | 0.394 | -1.860 – -0.315 | -2.759 | **.006** |
| Imperative | -0.251 | 0.238 | -0.718 – 0.217 | -1.051 | .293 |
| Informative Question | -0.222 | 0.201 | -0.615 – 0.171 | -1.108 | .268 |
| Pedagogical Question | -0.318 | 0.248 | -0.803 – 0.168 | -1.282 | .200 |
| Age^1^ | 0.013 | 0.056 | -0.097 – 0.123 | 0.229 | .819 |
| Offset^1^ | 4.059 | 0.223 | 3.620 – 4.497 | 18.159 | **<.001** |
| Novelty × Declarative | 0.304 | 0.215 | -0.118 – 0.726 | 1.413 | .158 |
| Novelty × Descriptive Question | 0.744 | 0.380 | -0.000 – 1.488 | 1.960 | .050 |
| Novelty × Directive Question | 3.609 | 1.121 | 1.411 – 5.807 | 3.218 | **.001** |
| Novelty × Imperative | 0.475 | 0.332 | -0.176 – 1.126 | 1.431 | .152 |
| Novelty × Informative Question | 0.374 | 0.275 | -0.165 – 0.914 | 1.360 | .174 |
| Novelty × Pedagogical Question | 0.083 | 0.345 | -0.593 – 0.759 | 0.240 | .810 |

*Note.* *SE* = standard error; CI = confidence interval. ^1^z-transformed

**Table S12**

*Model 2.1 Mothers only:* The role of topical alignment of parent speech in sustaining infant gaze

| *Predictors* | *Estimates* | *SE* | *95% CI* | *Statistic* | *p* |
| --- | --- | --- | --- | --- | --- |
| Intercept | 4539.055 | 749.845 | 3068.640 – 6009.470 | 6.053 | **<.001** |
| Alignment (misaligned) | -1097.527 | 448.781 | -1977.569 – -217.486 | -2.446 | **.015** |
| Novelty (familiar) | 390.962 | 660.204 | -903.671 – 1685.594 | 0.592 | .554 |
| Offset^1^ | 807.529 | 127.292 | 557.914 – 1057.144 | 6.344 | **<.001** |
| Age^1^ | 84.324 | 328.808 | -560.454 – 729.102 | 0.256 | .798 |

*Note.* *SE* = standard error; CI = confidence interval. ^1^z-transformed

**Table S13**

*Model 2.2 Mothers only:* The role of alignment, novelty and content of parent speech in sustaining infant gaze

| *Predictors* | *Estimates* | *SE* | *95% CI* | *Statistic* | *p* |
| --- | --- | --- | --- | --- | --- |
| Intercept | 4929.788 | 814.358 | 3332.863 – 6526.714 | 6.054 | **<.001** |
| Alignment (misaligned) | -1098.219 | 449.757 | -1980.174 – -216.263 | -2.442 | **.015** |
| Offset^1^ | 793.648 | 127.821 | 542.996 – 1044.300 | 6.209 | **<.001** |
| Novelty (familiar) | -282.929 | 782.190 | -1816.775 – 1250.916 | -0.362 | .718 |
| Referential | 95.299 | 382.147 | -654.076 – 844.674 | 0.249 | .803 |
| Social | 421.583 | 575.173 | -706.310 – 1549.476 | 0.733 | .464 |
| Situational | -644.192 | 417.077 | -1462.065 – 173.681 | -1.545 | .123 |
| Object Information | -773.997 | 421.317 | -1600.184 – 52.190 | -1.837 | .066 |
| Age^1^ | 61.656 | 327.832 | -581.210 – 704.521 | 0.188 | .851 |
| Novelty × Referential | -398.983 | 505.580 | -1390.406 – 592.440 | -0.789 | .430 |
| Novelty × Social | -1123.799 | 740.505 | -2575.902 – 328.304 | -1.518 | .129 |
| Novelty × Situational | 1156.773 | 554.163 | 70.081 – 2243.464 | 2.087 | **.037** |
| Novelty × Object Information | 1779.176 | 559.652 | 681.720 – 2876.632 | 3.179 | **.001** |

*Note.* *SE* = standard error; CI = confidence interval. ^1^z-transformed

**Table S14**

*Model 2.3 Mothers only:* The role of alignment, novelty and communicative intent of parent speech in sustaining infant gaze

| *Predictors* | *Estimates* | *SE* | *95% CI* | *Statistic* | *p* |
| --- | --- | --- | --- | --- | --- |
| Intercept | 4124.879 | 810.597 | 2535.328 – 5714.431 | 5.089 | **<.001** |
| Alignment (misaligned) | -1130.802 | 449.450 | -2012.156 – -249.447 | -2.516 | **.012** |
| Novelty (familiar) | 844.688 | 782.904 | -690.558 – 2379.934 | 1.079 | .281 |
| Declarative | 178.398 | 461.883 | -727.338 – 1084.135 | 0.386 | .699 |
| Descriptive Question | -501.459 | 902.772 | -2271.763 – 1268.844 | -0.555 | .579 |
| Directive Question | 3516.886 | 1390.563 | 790.044 – 6243.729 | 2.529 | **.011** |
| Imperative | 242.581 | 679.589 | -1090.068 – 1575.230 | 0.357 | .721 |
| Informative Question | 1723.243 | 593.220 | 559.960 – 2886.526 | 2.905 | **.004** |
| Pedagogical Question | 662.660 | 670.527 | -652.218 – 1977.538 | 0.988 | .323 |
| Age^1^ | 96.430 | 328.158 | -547.075 – 739.936 | 0.294 | .769 |
| Offset^1^ | 825.790 | 127.506 | 575.755 – 1075.826 | 6.476 | **<.001** |
| Novelty × Declarative | 222.718 | 616.355 | -985.931 – 1431.368 | 0.361 | .718 |
| Novelty × Descriptive Question | 365.038 | 1117.658 | -1826.649 – 2556.725 | 0.327 | .744 |
| Novelty × Directive Question | -4808.267 | 1802.424 | -8342.754 – -1273.781 | -2.668 | **.008** |
| Novelty × Imperative | -422.238 | 932.817 | -2251.458 – 1406.982 | -0.453 | .651 |
| Novelty × Informative Question | -2299.122 | 784.343 | -3837.190 – -761.054 | -2.931 | **.003** |
| Novelty × Pedagogical Question | -1033.431 | 960.029 | -2916.013 – 849.151 | -1.076 | .282 |

*Note.* *SE* = standard error; CI = confidence interval. ^1^z-transformed

**Table S15**

*Model 1.2 – Preliminary*

| Predictors | Estimate | *SE* | 95% CI | *z*-statistic | *p* |
| --- | --- | --- | --- | --- | --- |
| Intercept | 2.578 | 0.195 | 2.195 – 2.960 | 13.213 | **<.001** |
| Alignment (misaligned) | -0.371 | 0.243 | -0.847 – 0.105 | -1.528 | .126 |
| Novelty (familiar) | -0.394 | 0.241 | -0.867 – 0.080 | -1.630 | .103 |
| Offset^1^ | 3.682 | 0.187 | 3.316 – 4.048 | 19.736 | **<.001** |
| Age^1^ | -0.032 | 0.039 | -0.109 – 0.045 | -0.804 | .421 |
| Referential | -0.511 | 0.177 | -0.858 – -0.164 | -2.886 | **.004** |
| Social | -0.592 | 0.220 | -1.023 – -0.160 | -2.689 | **.007** |
| Situational | -0.279 | 0.199 | -0.669 – 0.111 | -1.402 | .161 |
| Object Information | -0.475 | 0.180 | -0.828 – -0.122 | -2.636 | **.008** |
| Alignment × Novelty | 0.042 | 0.334 | -0.611 – 0.696 | 0.127 | .899 |
| Alignment × Referential | 0.099 | 0.245 | -0.381 – 0.579 | 0.404 | .686 |
| Alignment × Social | -0.008 | 0.314 | -0.622 – 0.607 | -0.024 | .981 |
| Alignment × Situational | 0.178 | 0.272 | -0.354 – 0.710 | 0.657 | .511 |
| Alignment × Object Information | 0.182 | 0.251 | -0.310 – 0.674 | 0.725 | .468 |
| Novelty × Referential | 0.109 | 0.239 | -0.359 – 0.576 | 0.456 | .649 |
| Novelty × Social | 0.782 | 0.304 | 0.186 – 1.378 | 2.573 | **.010** |
| Novelty × Situational | 0.310 | 0.260 | -0.200 – 0.820 | 1.190 | .234 |
| Novelty × Object Information | 0.482 | 0.246 | 0.001 – 0.963 | 1.962 | **.050** |
| Alignment × Novelty × Referential | -0.041 | 0.334 | -0.696 – 0.614 | -0.122 | .903 |
| Alignment × Novelty × Social | -0.054 | 0.430 | -0.896 – 0.789 | -0.125 | .901 |
| Alignment × Novelty × Situational | -0.324 | 0.363 | -1.035 – 0.388 | -0.891 | .373 |
| Alignment × Novelty × Object Information | -0.253 | 0.346 | -0.931 – 0.425 | -0.732 | .464 |

*Note.* *SE* = standard error; CI = confidence interval. ^1^z-transformed

**Table S16**

*Model 2.1 – Preliminary*

| Predictors | Estimate | *SE* | 95% CI | *z*-statistic | *p* |
| --- | --- | --- | --- | --- | --- |
| Intercept | 4448.512 | 554.373 | 3361.491 – 5535.533 | 8.024 | **<.001** |
| Alignment (misaligned) | -861.954 | 411.802 | -1669.420 – -54.488 | -2.093 | **.036** |
| Novelty (familiar) | 127.272 | 639.081 | -1125.846 – 1380.390 | 0.199 | .842 |
| Offset^1^ | 754.917 | 112.998 | 533.348 – 976.485 | 6.681 | **<.001** |
| Age^1^ | -21.708 | 278.531 | -567.855 – 524.439 | -0.078 | .938 |
| Alignment × Novelty | -566.752 | 617.888 | -1778.314 – 644.811 | -0.917 | .359 |

*Note.* *SE* = standard error; CI = confidence interval. ^1^z-transformed

**Table S17**

*Model 2.2 – Preliminary*

| Predictors | Estimate | *SE* | 95% CI | *z*-statistic | *p* |
| --- | --- | --- | --- | --- | --- |
| Intercept | 4525.686 | 694.743 | 3163.423 – 5887.949 | 6.514 | **<.001** |
| Alignment (misaligned) | -672.245 | 755.122 | -2152.900 – 808.411 | -0.890 | .373 |
| Novelty (familiar) | -514.989 | 803.123 | -2089.764 – 1059.787 | -0.641 | .521 |
| Offset^1^ | 746.264 | 113.703 | 523.313 – 969.214 | 6.563 | **<.001** |
| Age^1^ | 1.849 | 281.401 | -549.926 – 553.623 | 0.007 | .995 |
| Referential | 465.847 | 447.384 | -411.391 – 1343.085 | 1.041 | .298 |
| Social | 129.937 | 649.820 | -1144.240 – 1404.114 | 0.200 | .842 |
| Situational | -359.768 | 497.304 | -1334.890 – 615.354 | -0.723 | .469 |
| Object Information | -630.222 | 484.930 | -1581.080 – 320.636 | -1.300 | .194 |
| Alignment × Novelty | -197.010 | 969.677 | -2098.368 – 1704.348 | -0.203 | .839 |
| Alignment × Referential | -458.597 | 688.959 | -1809.519 – 892.325 | -0.666 | .506 |
| Alignment × Social | 224.251 | 990.399 | -1717.739 – 2166.241 | 0.226 | .821 |
| Alignment × Situational | -289.147 | 748.626 | -1757.064 – 1178.770 | -0.386 | .699 |
| Alignment × Object information | -31.354 | 747.868 | -1497.786 – 1435.078 | -0.042 | .967 |
| Novelty × Referential | -524.822 | 580.196 | -1662.481 – 612.836 | -0.905 | .366 |
| Novelty × Social | -647.430 | 831.449 | -2277.750 – 982.889 | -0.779 | .436 |
| Novelty × Situational | 1170.446 | 643.996 | -92.311 – 2433.204 | 1.817 | .069 |
| Novelty × Object Information | 1836.072 | 637.188 | 586.665 – 3085.480 | 2.882 | **.004** |
| Alignment × Novelty × Referential | -74.061 | 917.400 | -1872.914 – 1724.791 | -0.081 | .936 |
| Alignment × Novelty × Social | -167.503 | 1290.568 | -2698.070 – 2363.064 | -0.130 | .897 |
| Alignment × Novelty × Situational | -253.639 | 999.442 | -2213.360 – 1706.083 | -0.254 | .800 |
| Alignment × Novelty × Object Information | -339.535 | 998.351 | -2297.118 – 1618.047 | -0.340 | .734 |

*Note.* *SE* = standard error; CI = confidence interval. ^1^z-transformed

**Table S18**

*Model 1.3 – Preliminary*

| Predictors | Estimate | *SE* | 95% CI | *z*-statistic | *p* |
| --- | --- | --- | --- | --- | --- |
| Intercept | 2.223 | 0.191 | 1.849 – 2.597 | 11.636 | **<.001** |
| Alignment (misaligned) | -0.336 | 0.233 | -0.794 – 0.121 | -1.440 | .150 |
| Offset^1^ | 3.668 | 0.186 | 3.304 – 4.033 | 19.725 | **<.001** |
| Age^1^ | -0.021 | 0.047 | -0.113 – 0.072 | -0.439 | .660 |
| Novelty (familiar) | -0.126 | 0.227 | -0.570 – 0.318 | -0.556 | .578 |
| Declarative | -0.343 | 0.211 | -0.758 – 0.071 | -1.624 | .104 |
| Imperative | 0.003 | 0.337 | -0.658 – 0.663 | 0.008 | .994 |
| Descriptive Question | -1.142 | 0.392 | -1.910 – -0.373 | -2.912 | **.004** |
| Directive Question | -1.358 | 0.546 | -2.428 – -0.288 | -2.488 | **.013** |
| Informative Question | -0.427 | 0.271 | -0.957 – 0.104 | -1.577 | .115 |
| Pedagogical Question | -0.185 | 0.334 | -0.839 – 0.470 | -0.553 | .580 |
| Alignment × Novelty | -0.252 | 0.313 | -0.866 – 0.362 | -0.805 | .421 |
| Alignment × Declarative | 0.088 | 0.289 | -0.477 – 0.654 | 0.305 | .760 |
| Alignment × Imperative | -0.137 | 0.460 | -1.038 – 0.764 | -0.298 | .766 |
| Alignment × Descriptive Question | 0.535 | 0.518 | -0.480 – 1.550 | 1.033 | .302 |
| Alignment × Directive Question | 0.445 | 0.723 | -0.973 – 1.863 | 0.615 | .539 |
| Alignment × Informative Question | 0.536 | 0.369 | -0.187 – 1.260 | 1.453 | .146 |
| Alignment × Pedagogical Question | 0.088 | 0.454 | -0.802 – 0.977 | 0.194 | .847 |
| Novelty × Declarative | 0.047 | 0.280 | -0.503 – 0.597 | 0.168 | .866 |
| Novelty × Imperative | 0.089 | 0.449 | -0.791 – 0.969 | 0.198 | .843 |
| Novelty × Descriptive Question | 0.971 | 0.517 | -0.042 – 1.984 | 1.879 | .060 |
| Novelty × Directive Question | 3.061 | 1.204 | 0.701 – 5.421 | 2.542 | **.011** |
| Novelty × Informative Question | 0.409 | 0.373 | -0.322 – 1.140 | 1.097 | .273 |
| Novelty × Pedagogical Question | -0.138 | 0.428 | -0.977 – 0.702 | -0.321 | .748 |
| Alignment × Novelty × Declarative | 0.140 | 0.391 | -0.627 – 0.906 | 0.357 | .721 |
| Alignment × Novelty × Imperative | 0.616 | 0.639 | -0.636 – 1.869 | 0.965 | .335 |
| Alignment × Novelty × Descriptive Question | -0.343 | 0.689 | -1.694 – 1.008 | -0.498 | .619 |
| Alignment × Novelty × Directive Question | -0.846 | 1.474 | -3.736 – 2.044 | -0.574 | .566 |
| Alignment × Novelty × Informative Question | -0.078 | 0.511 | -1.079 – 0.924 | -0.152 | .879 |
| Alignment × Novelty × Pedagogical Question | 0.145 | 0.639 | -1.107 – 1.397 | 0.227 | .821 |

*Note.* *SE* = standard error; CI = confidence interval. ^1^z-transformed

**Table S19**

*Model 2.3 – Preliminary*

| Predictors | Estimate | *SE* | 95% CI | *z*-statistic | *p* |
| --- | --- | --- | --- | --- | --- |
| Intercept | 4100.529 | 701.909 | 2724.213 – 5476.846 | 5.842 | **<.001** |
| Alignment (misaligned) | -936.992 | 760.390 | -2427.980 – 553.995 | -1.232 | .218 |
| Novelty (familiar) | 644.582 | 800.905 | -925.847 – 2215.012 | 0.805 | .421 |
| Offset^1^ | 763.920 | 113.400 | 541.563 – 986.277 | 6.737 | **<.001** |
| Age^1^ | 3.775 | 283.183 | -551.496 – 559.045 | 0.013 | .989 |
| Declarative | 261.648 | 537.805 | -792.890 – 1316.186 | 0.487 | .627 |
| Imperative | 138.497 | 830.514 | -1489.991 – 1766.985 | 0.167 | .868 |
| Descriptive Question | -299.306 | 1132.355 | -2519.648 – 1921.036 | -0.264 | .792 |
| Directive Question | 3481.813 | 1787.955 | -24.042 – 6987.669 | 1.947 | .052 |
| Informative Question | 1409.531 | 759.834 | -80.365 – 2899.426 | 1.855 | .064 |
| Pedagogical Question | 650.105 | 793.737 | -906.270 – 2206.479 | 0.819 | .413 |
| Alignment × Novelty | -632.631 | 946.693 | -2488.924 – 1223.662 | -0.668 | .504 |
| Alignment × Declarative | -424.928 | 821.568 | -2035.873 – 1186.017 | -0.517 | .605 |
| Alignment × Imperative | 441.351 | 1273.864 | -2056.465 – 2939.168 | 0.346 | .729 |
| Alignment × Descriptive Question | -140.001 | 1585.139 | -3248.171 – 2968.168 | -0.088 | .930 |
| Alignment × Directive Question | -1250.004 | 2536.751 | -6224.111 – 3724.102 | -0.493 | .622 |
| Alignment × Informative Question | 374.036 | 1062.984 | -1710.283 – 2458.355 | 0.352 | .725 |
| Alignment × Pedagogical Question | -490.326 | 1223.066 | -2888.537 – 1907.884 | -0.401 | .689 |
| Novelty × Declarative | -5.880 | 707.253 | -1392.675 – 1380.915 | -0.008 | .993 |
| Novelty × Imperative | 3.951 | 1104.646 | -2162.059 – 2169.962 | 0.004 | .997 |
| Novelty × Descriptive Question | -514.185 | 1381.936 | -3223.909 – 2195.540 | -0.372 | .710 |
| Novelty × Directive Question | -4928.968 | 2226.734 | -9295.190 – -562.747 | -2.214 | **.027** |
| Novelty × Informative Question | -2268.851 | 975.972 | -4182.555 – -355.147 | -2.325 | **.020** |
| Novelty × Pedagogical Question | -954.642 | 1048.830 | -3011.207 – 1101.923 | -0.910 | .363 |
| Alignment × Novelty × Declarative | 462.975 | 1105.532 | -1704.772 – 2630.723 | 0.419 | .675 |
| Alignment × Novelty × Imperative | -902.993 | 1749.612 | -4333.664 – 2527.677 | -0.516 | .606 |
| Alignment × Novelty × Descriptive Question | 1105.120 | 1972.577 | -2762.746 – 4972.986 | 0.560 | .575 |
| Alignment × Novelty × Directive Question | 2249.998 | 3303.581 | -4227.723 – 8727.720 | 0.681 | .496 |
| Alignment × Novelty × Informative Question | 152.301 | 1409.567 | -2611.603 – 2916.206 | 0.108 | .914 |
| Alignment × Novelty × Pedagogical Question | -481.188 | 1904.602 | -4215.766 – 3253.390 | -0.253 | .801 |

*Note.* *SE* = standard error; CI = confidence interval. ^1^z-transformed

**Table S20**

*Output of Omnibus Model 1:* Differences in the alignment, novelty, content, and communicative intent of parent speech in infant-led and parent-led interactions

| *Predictors* | *Log-Odds* | *SE* | *95% CI* | *z-statistic* | *p* |
| --- | --- | --- | --- | --- | --- |
| Intercept | 2.638 | 0.267 | 2.115 – 3.161 | 9.886 | **<.001** |
| Alignment (misaligned) | -0.286 | 0.078 | -0.439 – -0.133 | -3.665 | **<.001** |
| Novelty (familiar) | -0.508 | 0.326 | -1.147 – 0.132 | -1.556 | .120 |
| Referential | -0.485 | 0.129 | -0.737 – -0.232 | -3.762 | **<.001** |
| Social | -0.614 | 0.163 | -0.933 – -0.295 | -3.778 | **<.001** |
| Situational | -0.253 | 0.210 | -0.664 – 0.158 | -1.208 | .227 |
| Object Information | -0.368 | 0.128 | -0.619 – -0.117 | -2.869 | **.004** |
| Imperative | -0.224 | 0.300 | -0.811 – 0.363 | -0.747 | .455 |
| Declarative | -0.095 | 0.235 | -0.556 – 0.367 | -0.402 | .688 |
| Question | -0.050 | 0.252 | -0.544 – 0.444 | -0.199 | .842 |
| Offset^1^ | 3.697 | 0.187 | 3.330 – 4.065 | 19.730 | **<.001** |
| Age^1^ | -0.041 | 0.039 | -0.118 – 0.035 | -1.055 | .291 |
| Novelty × Referential | 0.125 | 0.175 | -0.218 – 0.468 | 0.714 | .475 |
| Novelty × Social | 0.762 | 0.220 | 0.331 – 1.194 | 3.463 | **.001** |
| Novelty × Situational | 0.256 | 0.270 | -0.274 – 0.786 | 0.947 | .344 |
| Novelty × Object Information | 0.359 | 0.178 | 0.011 – 0.708 | 2.024 | **.043** |
| Novelty × Imperative | 0.425 | 0.402 | -0.363 – 1.213 | 1.057 | .291 |
| Novelty × Declarative | -0.039 | 0.308 | -0.642 – 0.564 | -0.125 | .900 |
| Novelty × Question | 0.222 | 0.333 | -0.431 – 0.874 | 0.666 | .506 |

*Note.* *SE* = standard error; CI = confidence interval. ^1^z-transformed

Full model syntax:

*leader ~ alignment + novelty*(referential + social + situational + object information + imperative + declarative + question) + z.age + z.offset +* *(alignment + novelty || ID) + (alignment || object)*

Null model syntax:

*leader ~ z.age +* *(alignment + novelty || ID) + (alignment || object)*

The full-null model comparison was significant (Χ²=997.3, p <.001).

**Table S21**

*Output of Omnibus Model 2:* The role of alignment, novelty, content, and communicative intent of parent speech in sustaining infant gaze

| *Predictors* | *Estimates* | *SE* | *95% CI* | *Statistic* | *p* |
| --- | --- | --- | --- | --- | --- |
| Intercept | 4234.978 | 875.297 | 2518.682 – 5951.273 | 4.838 | **<.001** |
| Alignment (misaligned) | -1273.581 | 406.787 | -2071.215 – -475.946 | -3.131 | **.002** |
| Novelty (familiar) | 3.569 | 1017.001 | -1990.582 – 1997.721 | 0.004 | .997 |
| Referential | 170.411 | 360.521 | -536.503 – 877.326 | 0.473 | .636 |
| Social | -52.522 | 508.406 | -1049.413 – 944.368 | -0.103 | .918 |
| Situational | -25.826 | 579.906 | -1162.913 – 1111.261 | -0.045 | .964 |
| Object Information | -634.920 | 378.018 | -1376.143 – 106.304 | -1.680 | .093 |
| Imperative | 431.715 | 850.322 | -1235.610 – 2099.040 | 0.508 | .612 |
| Declarative | 314.702 | 636.099 | -932.571 – 1561.976 | 0.495 | .621 |
| Question | 1200.003 | 688.328 | -149.681 – 2549.687 | 1.743 | .081 |
| Age^1^ | -17.152 | 280.892 | -567.929 – 533.626 | -0.061 | .951 |
| Offset^1^ | 752.733 | 113.668 | 529.850 – 975.615 | 6.622 | **<.001** |
| Novelty × Referential | -350.803 | 473.367 | -1278.988 – 577.383 | -0.741 | .459 |
| Novelty × Social | -375.523 | 654.709 | -1659.287 – 908.242 | -0.574 | .566 |
| Novelty × Situational | 493.252 | 739.551 | -956.871 – 1943.375 | 0.667 | .505 |
| Novelty × Object Information | 1716.987 | 507.624 | 721.630 – 2712.344 | 3.382 | **.001** |
| Novelty × Imperative | -404.815 | 1113.517 | -2588.215 – 1778.585 | -0.364 | .716 |
| Novelty × Declarative | -131.595 | 823.132 | -1745.605 – 1482.415 | -0.160 | .873 |
| Novelty × Question | -1816.075 | 904.803 | -3590.226 – -41.925 | -2.007 | **.045** |

*Note.* *SE* = standard error; CI = confidence interval. ^1^z-transformed

Full model syntax:

*SA ~ alignment + novelty*(referential + social + situational + object information + imperative + declarative + question) + z.age + z.offset + (alignment + novelty | ID) + (alignment | object)*

Null model syntax:

*SA ~ z.age + (alignment + novelty | ID) + (alignment | object)*

The full-null model comparison was significant (Χ²=78.5, p <.001).

**Figure S1**

*Figure 5 full, including two extreme values*


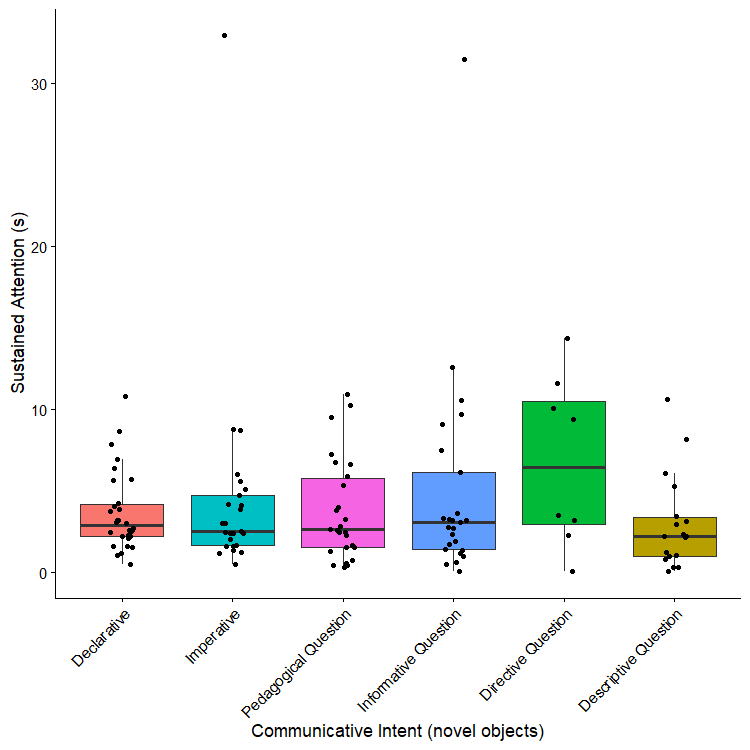


*Note.* The horizontal black lines mark the median and quartiles of the response variable. The points represent the raw data. The whiskers extend to the most extreme values within 1.5 IQR.

**Model Stability Results**

**Model 1.1:**


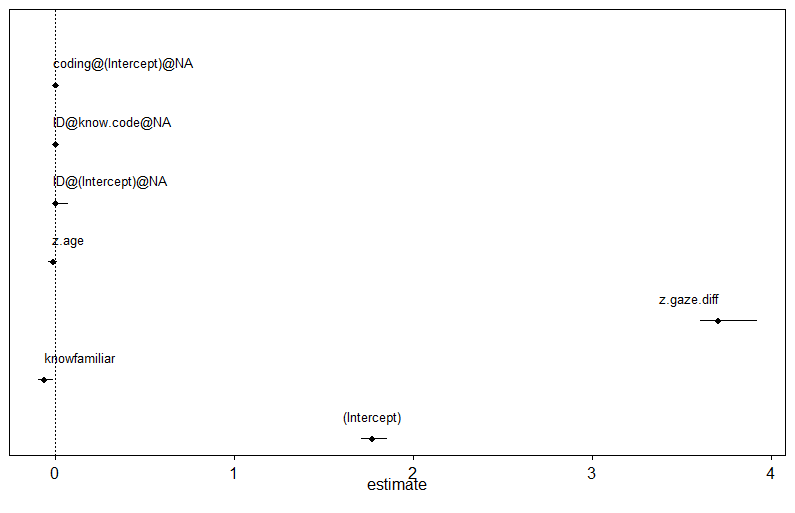


|  | *orig* | *min* | *max* |
| --- | --- | --- | --- |
| (Intercept) | 1.768 | 1.711 | 1.850 |
| knowfamiliar | -0.065 | -0.097 | -0.016 |
| z.gaze.diff | 3.703 | 3.602 | 3.918 |
| z.age | -0.013 | -0.040 | 0.006 |
| ID@(Intercept)@NA | 0.000 | 0.000 | 0.068 |
| ID@know.code@NA | 0.000 | 0.000 | 0.000 |
| coding@(Intercept)@NA | 0.000 | 0.000 | 0.000 |

Model complexity: 472.8571

**Model 1.2:**


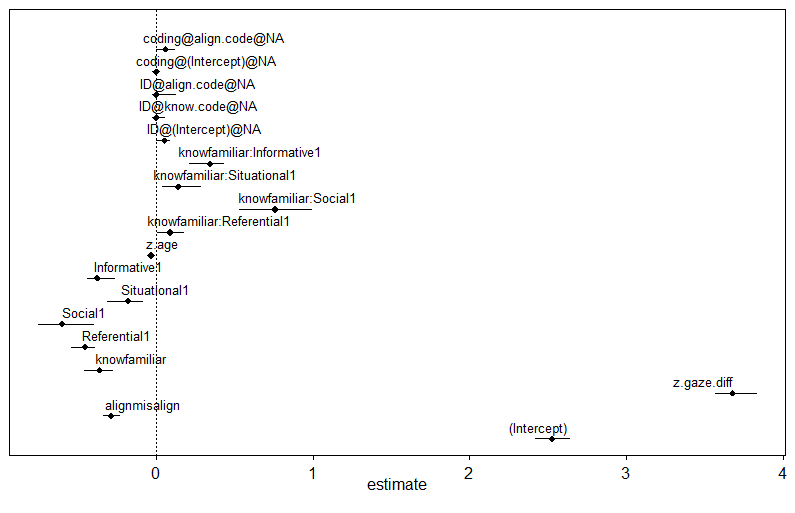


|  | *orig* | *min* | *max* |
| --- | --- | --- | --- |
| (Intercept) | 2.524 | 2.417 | 2.634 |
| alignmisalign | -0.288 | -0.334 | -0.239 |
| z.gaze.diff | 3.677 | 3.567 | 3.829 |
| knowfamiliar | -0.361 | -0.458 | -0.281 |
| Referential1 | -0.455 | -0.543 | -0.396 |
| Social1 | -0.600 | -0.753 | -0.404 |
| Situational1 | -0.181 | -0.312 | -0.087 |
| Informative1 | -0.375 | -0.439 | -0.267 |
| z.age | -0.034 | -0.048 | -0.011 |
| knowfamiliar:Referential1 | 0.088 | 0.006 | 0.175 |
| knowfamiliar:Social1 | 0.760 | 0.528 | 0.987 |
| knowfamiliar:Situational1 | 0.141 | 0.039 | 0.281 |
| knowfamiliar:Informative1 | 0.343 | 0.214 | 0.430 |
| ID@(Intercept)@NA | 0.054 | 0.000 | 0.082 |
| ID@know.code@NA | 0.000 | 0.000 | 0.050 |
| ID@align.code@NA | 0.000 | 0.000 | 0.120 |
| coding@(Intercept)@NA | 0.000 | 0.000 | 0.000 |
| coding@align.code@NA | 0.059 | 0.000 | 0.115 |

Model complexity: 232.222

**Model 1.2a (familiar):**


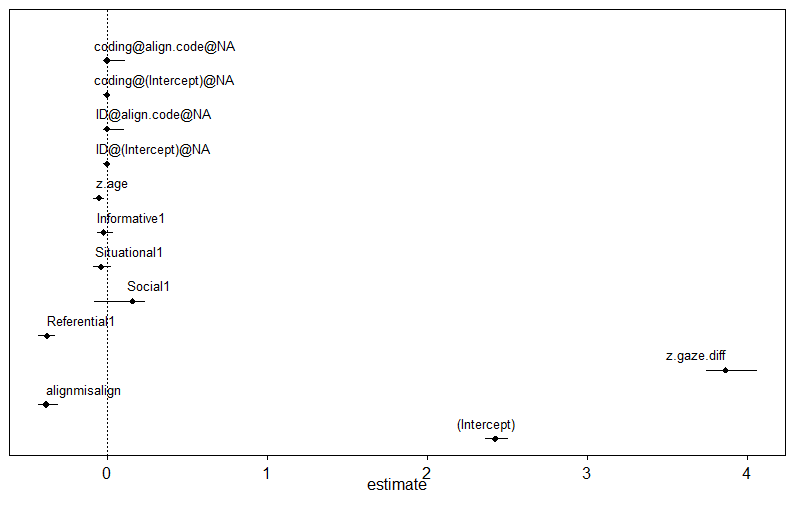


|  | *orig* | *min* | *max* |
| --- | --- | --- | --- |
| (Intercept) | 2.426 | 2.365 | 2.502 |
| alignmisalign | -0.382 | -0.426 | -0.310 |
| z.gaze.diff | 3.865 | 3.746 | 4.058 |
| Referential1 | -0.373 | -0.430 | -0.330 |
| Social1 | 0.161 | -0.078 | 0.235 |
| Situational1 | -0.038 | -0.083 | 0.022 |
| Informative1 | -0.022 | -0.061 | 0.032 |
| z.age | -0.049 | -0.087 | -0.021 |
| ID@(Intercept)@NA | 0.000 | 0.000 | 0.000 |
| ID@align.code@NA | 0.000 | 0.000 | 0.102 |
| coding@(Intercept)@NA | 0.000 | 0.000 | 0.000 |
| coding@align.code@NA | 0.000 | 0.000 | 0.111 |

Model complexity: 188.75

**Model 1.2b (novel):**


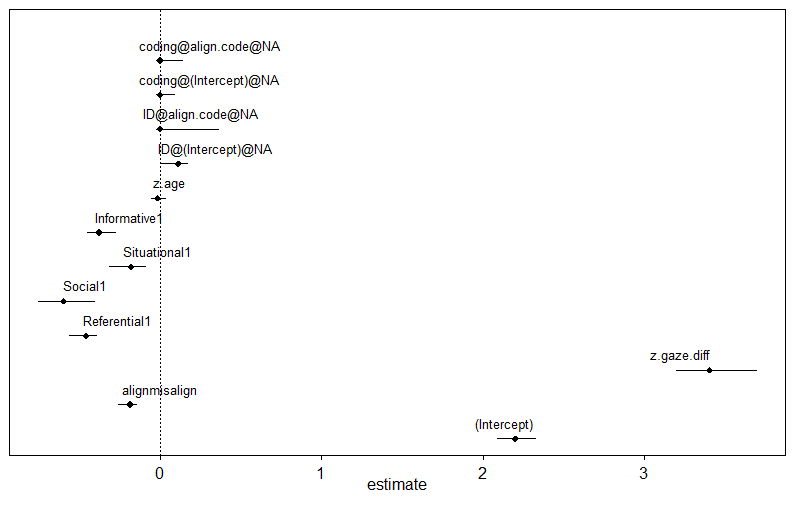


|  | *orig* | *min* | *max* |
| --- | --- | --- | --- |
| (Intercept) | 2.201 | 2.091 | 2.327 |
| alignmisalign | -0.185 | -0.260 | -0.149 |
| z.gaze.diff | 3.403 | 3.196 | 3.694 |
| Referential1 | -0.458 | -0.559 | -0.394 |
| Social1 | -0.598 | -0.755 | -0.407 |
| Situational1 | -0.181 | -0.316 | -0.093 |
| Informative1 | -0.377 | -0.451 | -0.274 |
| z.age | -0.014 | -0.051 | 0.030 |
| ID@(Intercept)@NA | 0.113 | 0.000 | 0.168 |
| ID@align.code@NA | 0.000 | 0.000 | 0.361 |
| coding@(Intercept)@NA | 0.000 | 0.000 | 0.089 |
| coding@align.code@NA | 0.000 | 0.000 | 0.141 |

Model complexity: 159.5833

**Model 1.3:**


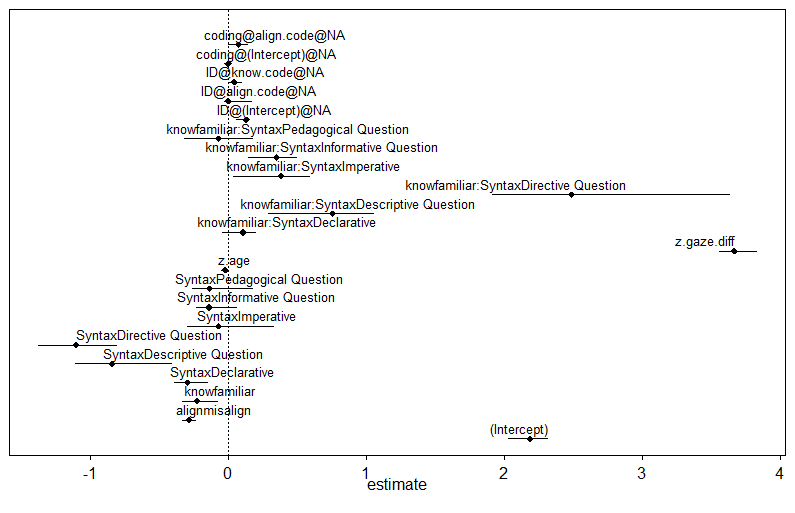


|  | *orig* | *min* | *max* |
| --- | --- | --- | --- |
| (Intercept) | 2.189 | 2.031 | 2.311 |
| alignmisalign | -0.285 | -0.332 | -0.237 |
| knowfamiliar | -0.228 | -0.335 | -0.081 |
| SyntaxDeclarative | -0.296 | -0.390 | -0.151 |
| SyntaxDescriptive Question | -0.843 | -1.106 | -0.415 |
| SyntaxDirective Question | -1.105 | -1.379 | -0.809 |
| SyntaxImperative | -0.069 | -0.298 | 0.323 |
| SyntaxInformative Question | -0.141 | -0.231 | 0.058 |
| SyntaxPedagogical Question | -0.135 | -0.262 | 0.174 |
| z.age | -0.022 | -0.039 | 0.001 |
| z.gaze.diff | 3.669 | 3.560 | 3.829 |
| knowfamiliar:SyntaxDeclarative | 0.105 | -0.045 | 0.192 |
| knowfamiliar:SyntaxDescriptive Question | 0.757 | 0.289 | 1.051 |
| knowfamiliar:SyntaxDirective Question | 2.488 | 1.911 | 3.631 |
| knowfamiliar:SyntaxImperative | 0.381 | 0.035 | 0.588 |
| knowfamiliar:SyntaxInformative Question | 0.351 | 0.143 | 0.491 |
| knowfamiliar:SyntaxPedagogical Question | -0.069 | -0.321 | 0.173 |
| ID@(Intercept)@NA | 0.130 | 0.060 | 0.148 |
| ID@align.code@NA | 0.000 | 0.000 | 0.167 |
| ID@know.code@NA | 0.044 | 0.000 | 0.097 |
| coding@(Intercept)@NA | 0.000 | 0.000 | 0.005 |
| coding@align.code@NA | 0.076 | 0.000 | 0.139 |

Model complexity: 190

**Model 1.3a (familiar):**


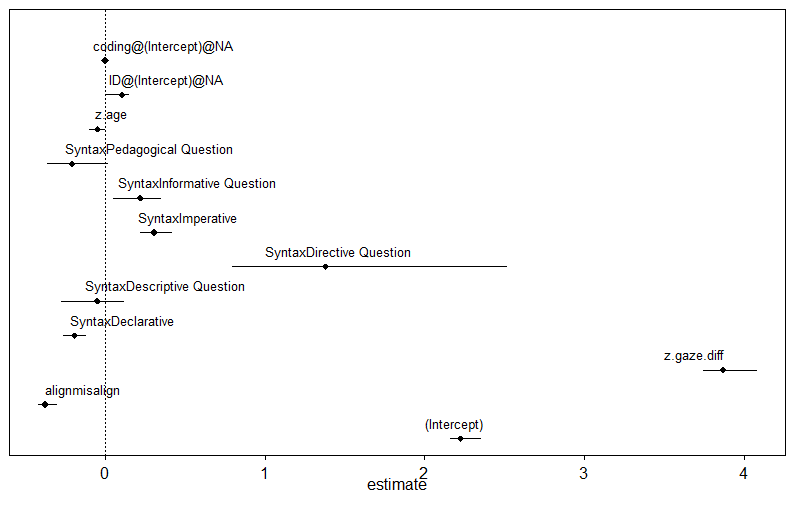


|  | *orig* | *min* | *max* |
| --- | --- | --- | --- |
| (Intercept) | 2.224 | 2.160 | 2.349 |
| alignmisalign | -0.378 | -0.421 | -0.306 |
| z.gaze.diff | 3.869 | 3.746 | 4.079 |
| SyntaxDeclarative | -0.192 | -0.265 | -0.126 |
| SyntaxDescriptive Question | -0.051 | -0.279 | 0.113 |
| SyntaxDirective Question | 1.381 | 0.796 | 2.509 |
| SyntaxImperative | 0.304 | 0.216 | 0.411 |
| SyntaxInformative Question | 0.219 | 0.051 | 0.347 |
| SyntaxPedagogical Question | -0.210 | -0.361 | 0.010 |
| z.age | -0.048 | -0.098 | -0.005 |
| ID@(Intercept)@NA | 0.104 | 0.000 | 0.146 |
| coding@(Intercept)@NA | 0.000 | 0.000 | 0.000 |

Model complexity: 188.75

**Model 1.3b (novel):**


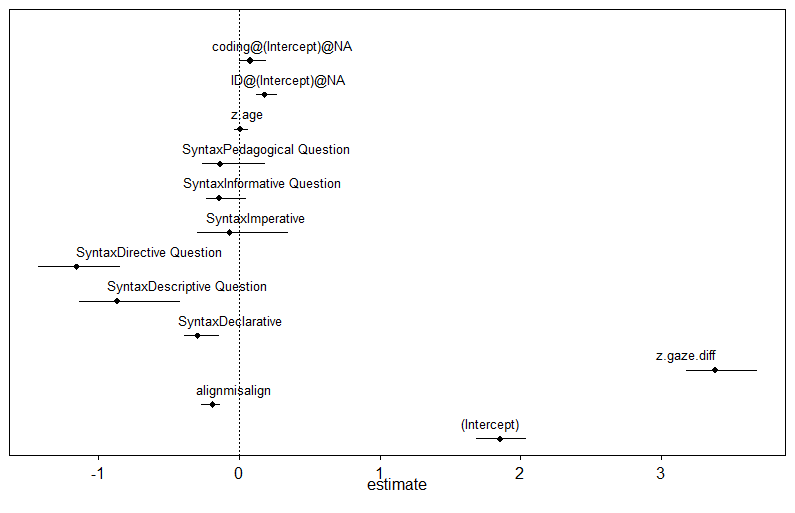


|  | *orig* | *min* | *max* |
| --- | --- | --- | --- |
| (Intercept) | 1.854 | 1.685 | 2.031 |
| alignmisalign | -0.186 | -0.265 | -0.141 |
| z.gaze.diff | 3.378 | 3.174 | 3.673 |
| SyntaxDeclarative | -0.293 | -0.386 | -0.144 |
| SyntaxDescriptive Question | -0.865 | -1.134 | -0.427 |
| SyntaxDirective Question | -1.152 | -1.426 | -0.846 |
| SyntaxImperative | -0.065 | -0.294 | 0.344 |
| SyntaxInformative Question | -0.142 | -0.231 | 0.047 |
| SyntaxPedagogical Question | -0.135 | -0.260 | 0.183 |
| z.age | 0.007 | -0.032 | 0.058 |
| ID@(Intercept)@NA | 0.181 | 0.126 | 0.268 |
| coding@(Intercept)@NA | 0.078 | 0.000 | 0.190 |

Model complexity: 159.5833

**Model 2.1:**


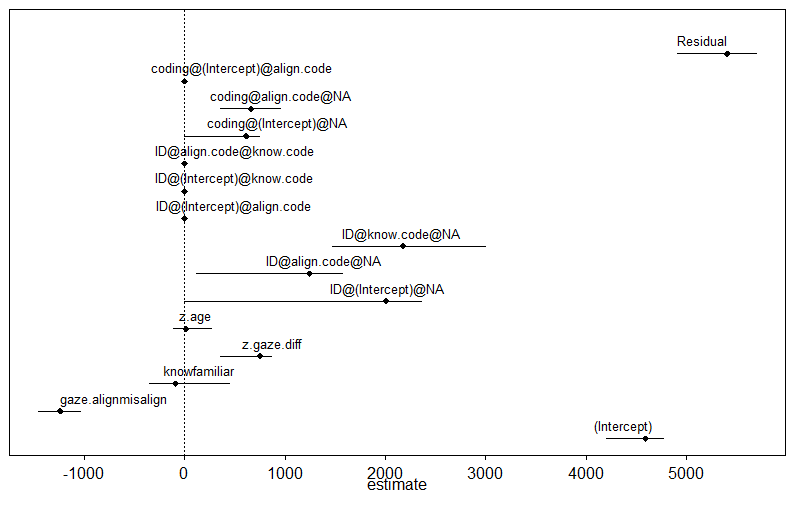


|  | *orig* | *min* | *max* |
| --- | --- | --- | --- |
| (Intercept) | 4595.221 | 4202.739 | 4770.130 |
| gaze.alignmisalign | -1240.593 | -1462.401 | -1039.671 |
| knowfamiliar | -93.018 | -349.355 | 444.426 |
| z.gaze.diff | 752.105 | 354.158 | 862.041 |
| z.age | 10.577 | -116.641 | 259.956 |
| ID@(Intercept)@NA | 2007.506 | 0.000 | 2358.950 |
| ID@align.code@NA | 1244.597 | 119.894 | 1566.557 |
| ID@know.code@NA | 2178.313 | 1471.904 | 2999.589 |
| ID@(Intercept)@align.code | -0.279 | -1.000 | -0.021 |
| ID@(Intercept)@know.code | -0.712 | -0.930 | -0.209 |
| ID@align.code@know.code | 0.224 | -0.141 | 0.826 |
| coding@(Intercept)@NA | 613.790 | 0.000 | 746.613 |
| coding@align.code@NA | 660.112 | 350.726 | 953.231 |
| coding@(Intercept)@align.code | 0.184 | -0.873 | 1.000 |
| Residual | 5410.685 | 4912.198 | 5702.055 |

Model complexity: 187.8667

**Model 2.2:**


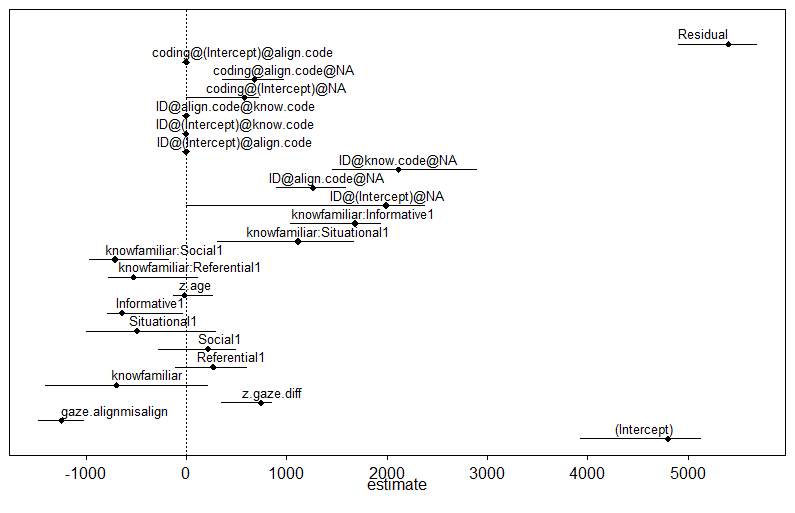


|  | *orig* | *min* | *max* |
| --- | --- | --- | --- |
| (Intercept) | 4799.336 | 3923.924 | 5117.431 |
| gaze.alignmisalign | -1243.025 | -1475.956 | -1027.467 |
| z.gaze.diff | 743.965 | 345.454 | 851.162 |
| knowfamiliar | -692.683 | -1407.296 | 213.107 |
| Referential1 | 268.592 | -106.044 | 602.597 |
| Social1 | 218.107 | -279.139 | 492.005 |
| Situational1 | -488.963 | -999.869 | 294.017 |
| Informative1 | -639.654 | -784.460 | -35.396 |
| z.age | -14.824 | -125.770 | 257.123 |
| knowfamiliar:Referential1 | -523.210 | -781.186 | 114.717 |
| knowfamiliar:Social1 | -708.955 | -968.858 | -183.780 |
| knowfamiliar:Situational1 | 1113.159 | 306.254 | 1664.000 |
| knowfamiliar:Informative1 | 1682.016 | 1040.128 | 1933.130 |
| ID@(Intercept)@NA | 1992.551 | 0.000 | 2373.457 |
| ID@align.code@NA | 1261.394 | 895.740 | 1588.277 |
| ID@know.code@NA | 2115.802 | 1457.282 | 2885.467 |
| ID@(Intercept)@align.code | -0.275 | -0.447 | -0.001 |
| ID@(Intercept)@know.code | -0.718 | -0.923 | -0.237 |
| ID@align.code@know.code | 0.236 | -0.135 | 0.672 |
| coding@(Intercept)@NA | 580.039 | 0.000 | 714.545 |
| coding@align.code@NA | 679.010 | 355.851 | 964.717 |
| coding@(Intercept)@align.code | 0.228 | -0.844 | 1.000 |
| Residual | 5403.736 | 4903.045 | 5681.675 |

Model complexity: 122.5217

**Model 2.2a (familiar):**


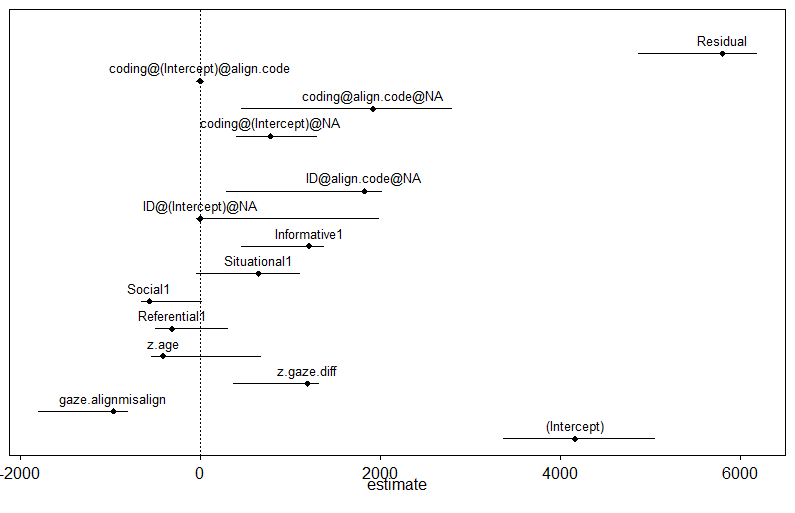


|  | *orig* | *min* | *max* |
| --- | --- | --- | --- |
| (Intercept) | 4163.222 | 3365.970 | 5043.594 |
| gaze.alignmisalign | -959.978 | -1799.984 | -809.802 |
| z.gaze.diff | 1192.887 | 367.001 | 1307.169 |
| z.age | -414.883 | -548.531 | 670.275 |
| Referential1 | -311.826 | -499.106 | 298.181 |
| Social1 | -561.289 | -649.881 | 12.168 |
| Situational1 | 648.025 | -46.919 | 1094.532 |
| Informative1 | 1206.871 | 460.201 | 1368.519 |
| ID@(Intercept)@NA | 0.000 | 0.000 | 1977.936 |
| ID@align.code@NA | 1825.581 | 294.013 | 2009.707 |
| ID@(Intercept)@align.code | NaN | -1.000 | 1.000 |
| coding@(Intercept)@NA | 781.041 | 401.660 | 1288.103 |
| coding@align.code@NA | 1917.468 | 460.365 | 2790.742 |
| coding@(Intercept)@align.code | 0.383 | -0.742 | 1.000 |
| Residual | 5801.078 | 4860.561 | 6176.058 |

Model complexity: 102.933

**Model 2.2b (novel):**


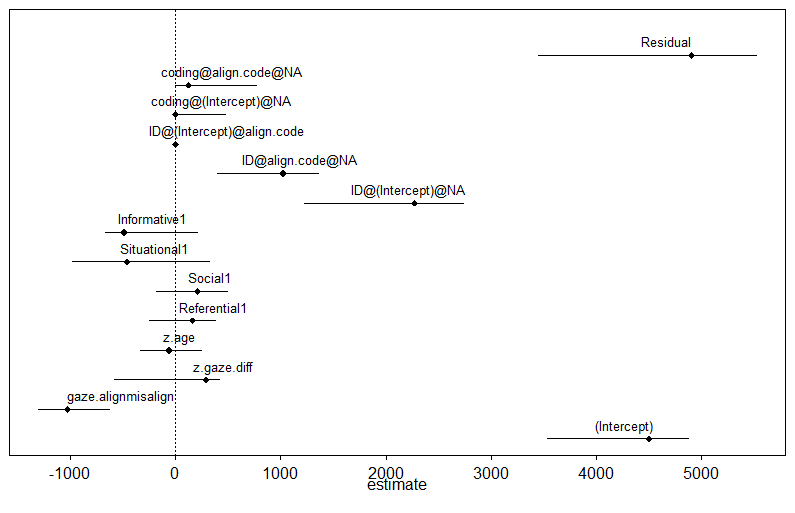


|  | *orig* | *min* | *max* |
| --- | --- | --- | --- |
| (Intercept) | 4505.291 | 3539.053 | 4881.799 |
| gaze.alignmisalign | -1028.011 | -1308.841 | -636.367 |
| z.gaze.diff | 288.999 | -582.894 | 411.505 |
| z.age | -65.400 | -341.380 | 247.737 |
| Referential1 | 162.705 | -251.328 | 379.944 |
| Social1 | 208.347 | -187.655 | 487.384 |
| Situational1 | -463.109 | -986.910 | 317.933 |
| Informative1 | -492.334 | -671.115 | 203.895 |
| ID@(Intercept)@NA | 2275.115 | 1222.118 | 2739.527 |
| ID@align.code@NA | 1019.926 | 393.199 | 1354.775 |
| ID@(Intercept)@align.code | -0.345 | -1.000 | 0.313 |
| coding@(Intercept)@NA | 0.000 | 0.000 | 474.069 |
| coding@align.code@NA | 124.352 | 0.000 | 770.463 |
| Residual | 4909.869 | 3448.870 | 5526.378 |

Model complexity: 91

**Model 2.3:**


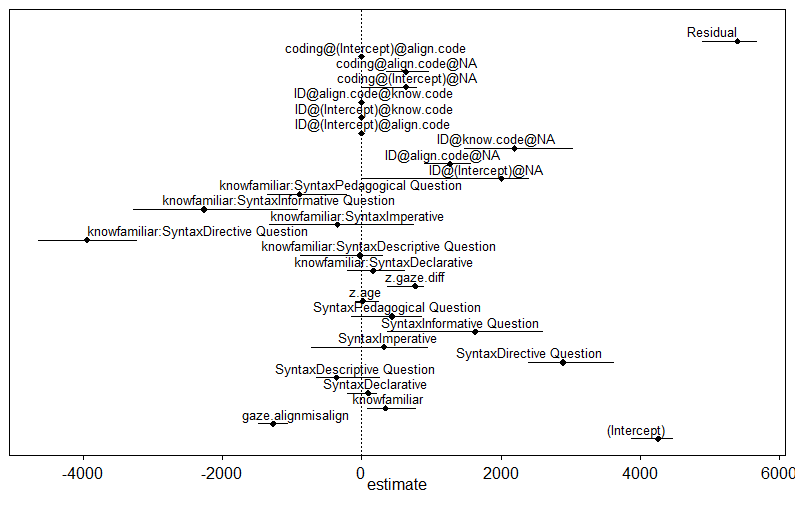


|  | *orig* | *min* | *max* |
| --- | --- | --- | --- |
| (Intercept) | 4257.572 | 3868.271 | 4458.820 |
| gaze.alignmisalign | -1270.697 | -1487.375 | -1073.271 |
| knowfamiliar | 344.931 | 75.775 | 773.090 |
| SyntaxDeclarative | 94.948 | -214.041 | 215.639 |
| SyntaxDescriptive Question | -359.379 | -647.678 | 245.590 |
| SyntaxDirective Question | 2888.566 | 2385.172 | 3608.452 |
| SyntaxImperative | 320.205 | -727.730 | 939.619 |
| SyntaxInformative Question | 1629.419 | 366.835 | 2592.029 |
| SyntaxPedagogical Question | 438.496 | -156.603 | 856.005 |
| z.age | 17.068 | -89.586 | 244.798 |
| z.gaze.diff | 768.594 | 368.002 | 888.797 |
| knowfamiliar:SyntaxDeclarative | 166.049 | -207.004 | 604.116 |
| knowfamiliar:SyntaxDescriptive Question | -26.032 | -884.789 | 290.035 |
| knowfamiliar:SyntaxDirective Question | -3944.046 | -4646.926 | -3243.126 |
| knowfamiliar:SyntaxImperative | -345.933 | -1333.560 | 746.482 |
| knowfamiliar:SyntaxInformative Question | -2265.797 | -3281.020 | -920.428 |
| knowfamiliar:SyntaxPedagogical Question | -892.518 | -1357.883 | -222.372 |
| ID@(Intercept)@NA | 2010.391 | 0.000 | 2395.484 |
| ID@align.code@NA | 1265.781 | 895.998 | 1564.116 |
| ID@know.code@NA | 2196.357 | 1476.334 | 3023.319 |
| ID@(Intercept)@align.code | -0.294 | -0.452 | -0.041 |
| ID@(Intercept)@know.code | -0.709 | -0.930 | -0.207 |
| ID@align.code@know.code | 0.229 | -0.125 | 0.667 |
| coding@(Intercept)@NA | 637.844 | 0.000 | 783.336 |
| coding@align.code@NA | 633.772 | 346.557 | 957.509 |
| coding@(Intercept)@align.code | 0.161 | -0.880 | 1.000 |
| Residual | 5399.479 | 4889.111 | 5668.157 |

Model complexity: 104.3704

**Model 2.3a (familiar):**


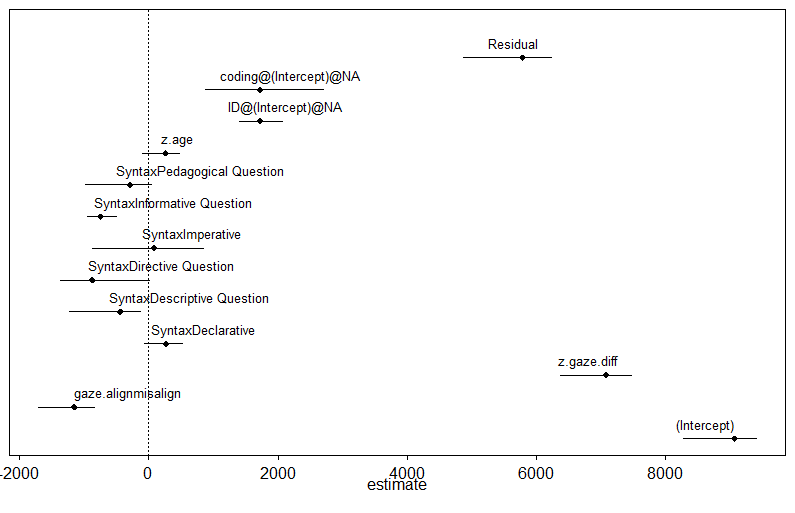


|  | *orig* | *min* | *max* |
| --- | --- | --- | --- |
| (Intercept) | 9080.912 | 8289.196 | 9419.226 |
| gaze.alignmisalign | -1155.453 | -1714.974 | -850.530 |
| z.gaze.diff | 7084.159 | 6376.155 | 7479.299 |
| SyntaxDeclarative | 268.739 | -69.958 | 512.378 |
| SyntaxDescriptive Question | -440.418 | -1235.636 | -133.041 |
| SyntaxDirective Question | -876.507 | -1370.462 | 8.599 |
| SyntaxImperative | 80.539 | -868.490 | 848.881 |
| SyntaxInformative Question | -747.951 | -948.410 | -497.833 |
| SyntaxPedagogical Question | -289.418 | -986.066 | 45.220 |
| z.age | 260.889 | -98.948 | 474.100 |
| ID@(Intercept)@NA | 1724.079 | 1405.530 | 2070.698 |
| coding@(Intercept)@NA | 1726.383 | 878.124 | 2702.146 |
| Residual | 5792.111 | 4880.315 | 6234.406 |

Model complexity: 118.7692

**Model 2.3b (novel):**


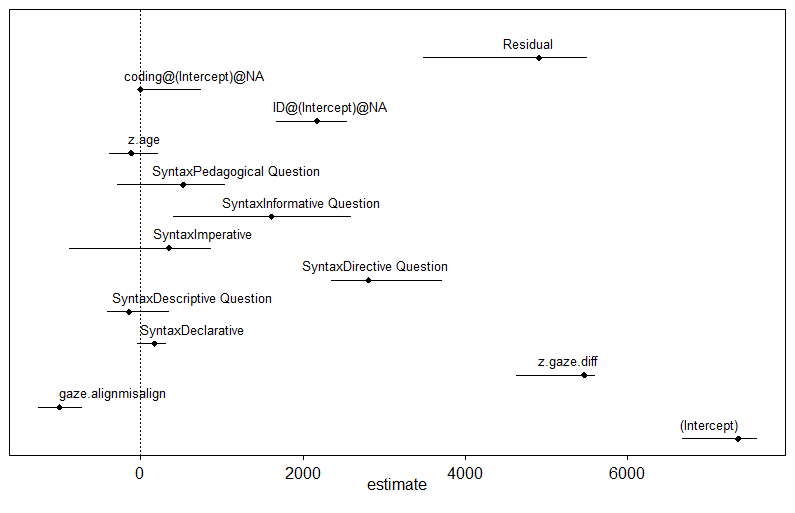


|  | *orig* | *min* | *max* |
| --- | --- | --- | --- |
| (Intercept) | 7361.931 | 6669.162 | 7584.740 |
| gaze.alignmisalign | -997.679 | -1261.801 | -736.224 |
| z.gaze.diff | 5463.892 | 4632.657 | 5582.252 |
| SyntaxDeclarative | 173.743 | -41.225 | 300.220 |
| SyntaxDescriptive Question | -142.389 | -411.932 | 342.943 |
| SyntaxDirective Question | 2808.035 | 2348.211 | 3708.941 |
| SyntaxImperative | 351.358 | -872.894 | 857.395 |
| SyntaxInformative Question | 1615.204 | 401.885 | 2578.497 |
| SyntaxPedagogical Question | 526.883 | -290.269 | 1027.419 |
| z.age | -114.522 | -386.961 | 201.824 |
| ID@(Intercept)@NA | 2171.004 | 1673.781 | 2533.091 |
| coding@(Intercept)@NA | 0.000 | 0.000 | 740.293 |
| Residual | 4905.821 | 3483.577 | 5492.527 |

Model complexity: 98
